# Two novel human anti-CD25 antibodies with antitumor activity inversely related to their affinity and in vitro activity

**DOI:** 10.1038/s41598-021-02449-y

**Published:** 2021-11-25

**Authors:** Deyong Song, Xiu Liu, Chuangchuang Dong, Qiaoping Wang, Chunjie Sha, Chuan Liu, Zhenfei Ning, Jing Han, Hong Liu, Mengqi Zong, Yanyan Zhao, Ying Li, Guangsheng Liu, Xin Shao, Changlin Dou

**Affiliations:** 1 Antibody Research and Development Center, Shandong Boan Biotechnology Co., Ltd., Yantai, 264670 People’s Republic of China; 2 State Key Laboratory of Long-Acting and Targeting Drug Delivery System, Shandong Luye Pharmaceutical Co. Ltd, Yantai, 264670 People’s Republic of China; 3 Shuimu BioSciences Ltd., Beijing, 102206 People’s Republic of China

**Keywords:** Immunotherapy, Cancer microenvironment, Antibody therapy

## Abstract

High tumor regulatory T (Treg) cell infiltration is associated with poor prognosis of many cancers. CD25 is highly expressed on tumor Treg cells and is a potential target for Treg deletion. Previously characterized anti-CD25 antibodies appear to have limited efficacy in tumor inhibition. Here we identified two human anti-CD25 antibodies, BA9 and BT942, which did not prevent the activation of IL-2R signaling pathway by IL-2. BT942 had weaker binding and cytotoxic activity to human CD25-expressing cell lines than BA9. But both demonstrated significant tumor growth inhibition in early and late-stage animal cancer models. BT942 resulted in a higher expansion of CD8^+^ T cells and CD4^+^ T cells in tumor microenvironment in mouse MC38 model compared to BA9. BT942 also demonstrated significant higher tumor growth inhibition and higher expansion of CD8^+^ T cells and CD4^+^ T cells in combination with an anti-PD1 antibody. Pharmacokinetic study of BT942 in cynomolgus monkeys demonstrated a half-life of 206.97 ± 19.03 h. Structural analysis by cryo-EM revealed that BT942 recognizes an epitope on opposite side of the CD25-IL-2 binding site, consistent with no IL-2 signaling blockade in vitro. BT942 appears to be an excellent candidate for cancer immunotherapy.

## Introduction

In human, regulatory T (Treg) cell population accounts for only 5% of CD4^+^ T cells, which are characterized by constitutively high expression of human CD25 (interleukin-2 receptor alpha, IL-2Ra) and immune suppression^1,2^. There are two subgroups of Tregs: the naturally occurring Treg cells (nTregs) and the inducible or adaptive Tregs (iTreg, Tr1). nTregs and iTregs mediate their suppression via cell contact-dependent mechanisms or through the production of soluble factors, such as TGF-beta, IL-10 and adenosine^3,4^. Removal of CD25^+^CD4^+^ T cells cause several autoimmune diseases in mice^5,6^.

The number of Treg cells is higher in peripheral blood mononuclear cells (PBMC) and tumors of many cancer patients, especially in tumors^7–11^. Treg cells can suppress most immune cells including CD4^+^ and CD8^+^T cells, B cells, NK cells, NKT cells and APCs, such as DCs, monocytes and macrophages^3,4^. High Treg infiltration is related to the poor prognosis of most solid tumors, such as cervical, ovarian, renal, melanomas, pancreatic, hepatocellular, gastric and breast cancers^12–18^. Recent systematic review and meta-analysis on FoxP3^+^ Treg cells revealed that prognostic role of FoxP3^+^ Tregs was highly influenced by tumor site and was also correlated with the molecular subtype and tumor stage^12^. Removing CD25^+^CD4^+^ T cells or in vivo administration of anti-CD25 monoclonal antibody in mice can induce tumor immunity or tumor suppression^19–21^. Consequently, Treg deletion from the tumor should be beneficial for tumor treatment. Removing Tregs is likely to increase the response rate of current immunotherapy by relieving Treg cell inhibition on effector T (T_eff_) cells, B cells and NK cells in the tumor microenvironment.

There are several targets on Treg cells. In addition to antibodies targeting CD25, Smyth and colleagues^22^ revealed that antibodies against other targets such as CTLA4, OX40 and GITR may facilitate the elimination of regulatory T cells in tumor microenvironment by effector functions of the antibody^22–25^. Such antibody-mediated killing of regulatory T cells may be more important than the antibody-mediated activation of effector T cells for the anti-tumor activities of those antibodies. However, among those targets, CD25 is expressed at high level^26^.

Although in vitro studies have confirmed that CD25 is transiently upregulated after T_eff_ cells are activated^27^, the studies in mouse models show that both the expression percentage and the level of expression of mouse CD25 in T_eff_ cells are much lower than Treg cells in tumor^19^. In human cancers, human CD25 is mainly expressed on CD4^+^FoxP3^+^ Treg cells and in all tumor types studied, the level of CD25 expression in CD4^+^FoxP3^+^ Treg cells is also significantly higher than that in CD4^+^FoxP3^−^ and CD8^+^ T cells^26^.

Several anti-CD25 antibodies had been developed. Anti-mouse CD25 monoclonal antibodies (clone PC61, rat IgG1) can only be effective when injected before tumor inoculation or early tumor establishment. Rat IgG1 can engage inhibitory Fcγ receptors FcγRIIb and activatory receptors FcγRIII, but not FcγRI and FcγRIV in mice. Specifically, in mice MOPC-70A models, it can only be effective when administered before day 2 after tumor inoculation^20^. In the mouse A20 model, anti-mouse CD25 monoclonal antibodies (PC61) could not inhibit tumor growth when administered at a time the tumor was palpable^19^. Arce Vargas and colleagues^26^ analyzed why the anti-CD25(clone PC61, rat IgG1) has only limited effects in mice. Their results shown that the high expression of FcRIIb and inappropriate Fc of anti-CD25 resulted in poor clearance of Treg in the tumor. Fc-optimized anti-CD25 (clone PC61, murine IgG2a) showed good efficacy in the established MC38, MCA205, CT26 models only in combination with anti-mouse PD1, but the efficacy of single anti-CD25 administration was weak^26^. The anti-mouse CD25 antibody (clone PC61) can block IL-2 signaling^28^ just as two anti-human CD25 antibodies in the clinic, basiliximab and daclizumab, which were used for multiple sclerosis or acute organ rejection through blocking IL-2 signaling^29,30^. As CD25 is also transiently expressed on a small population of activated T_eff_ cells, IL-2 can stimulate the effector T cells through the IL-2 receptor complex to facilitate tumor suppression. Anti-CD25 mAb that does not interfere with IL-2 downstream signaling should have better antitumor efficacy. The therapeutic potential of targeting CD25 led us to develop a screening strategy to identify potent human antibodies against CD25. Using immunized human antibody transgenic mice and phage display technologies we identified two human monoclonal antibodies, BA9 and BT942, that target CD25. In addition, we provide evidence that both BA9 and BT942 display significant binding activity to CD25 and that they do not prevent the activation of IL-2 downstream signaling pathway. Furthermore, we show that BA9 and BT942 have significant cell-mediated cytotoxicity and tumor suppression in both early phase and late phase of tumor establishment. Finally, we provide evidence that BT942 has the ability to synergize with PD1 inhibitor for cancer therapy.

## Results

### Phage display screening identifies human CD25-specific antibody candidates BT942 and BA9

To identify potential CD25 targeting antibodies, immunized human antibody transgenic mice were used in conjunction with phage display. Here human antibody transgenic mice were immunized with human CD25 protein. Titers of antibodies in the mice serum were tested by ELISA (enzyme-linked immunosorbent assay). Three days after the last immunization, the spleen was harvested from each mouse and used for the construction of the phage libraries. For these plates coated with CD25 or streptavidin-magnetic beads binding biotinylatedCD25 were used to capture phage of interest. Enriched phages were used to infect *E. coli* TG1 for expressing single-chain variable fragments (scFvs) and the binding and blocking activity were tested by ELISA. Clones that bind CD25 without blocking IL-2 were sequenced and converted to human IgG1 for in vitro evaluation. These analyses obtained two candidates BT942 and BA9 (Supplementary Fig. S1).

### Monoclonal antibodies BA9 and BT942 do not prevent the activation of IL-2R signaling pathway by IL-2

We examined if the binding of BA9 and BT942 to human CD25 alters IL-2 signaling by analyzing the phosphorylation of STAT5 in PBMCs by flow cytometry. The gating strategy for the flow cytometry is shown in Supplementary Fig. S2.

**Figure 1:**
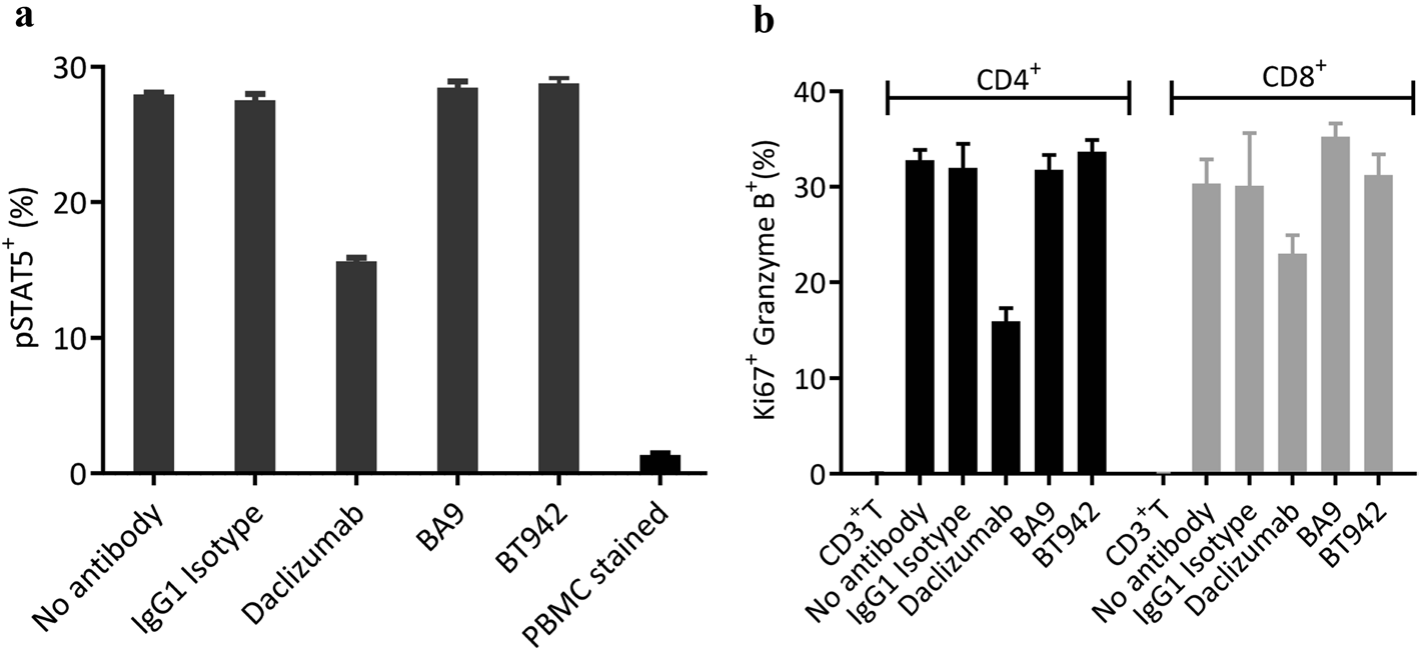
IL-2/IL-2 receptor blocking activity of BA9, BT942 and Daclizumab. (**a**) Characterization of anti-CD25 antibodies compared to isotype antibody in respect to blocking IL-2 signaling in a STAT5 phosphorylation assay using human PBMCs. PBMCs were co-cultured with 10 μg/mL antibody for 30 min, then 10 U/mL IL2 was added and cultured for 10 min. Isotype is a human IgG1 with kappa light chain (Crown Bio, C0001-2). Data were presented as mean ± SEM. (**b**) BA9 and BT942 do not alter activation and proliferation of CD8^+^ and CD4^+^ T cells in vitro IL-2 signaling by T cell activation assay. Antibodies were tested for 72 h at 10 µg/mL. Data were presented as mean ± SEM.

The data in Fig. 1a show that BA9 and BT942 do not block the activation of IL-2R signaling pathway by IL-2 as assessed through the percentage phosphorylation of STAT5 in PBMC by flow cytometry, whereas daclizumab significantly affect IL-2 signaling consistent with its mechanism. Unlike daclizumab, BA9 and BT942 also do not alter activation and proliferation of CD8^+^ and CD4^+^ T cells with Granzyme B and Ki67 expression as markers in vitro IL-2 signaling by T cell activation assay (Fig. 1b).

### BT942 displayed weaker binding activity than BA9 to human or cynomolgus CD25

To assess the binding activity of antibody candidates BT942 and BA9 to CD25, flow cytometry and surface plasmon resonance (SPR) analysis were used.

**Figure 2:**
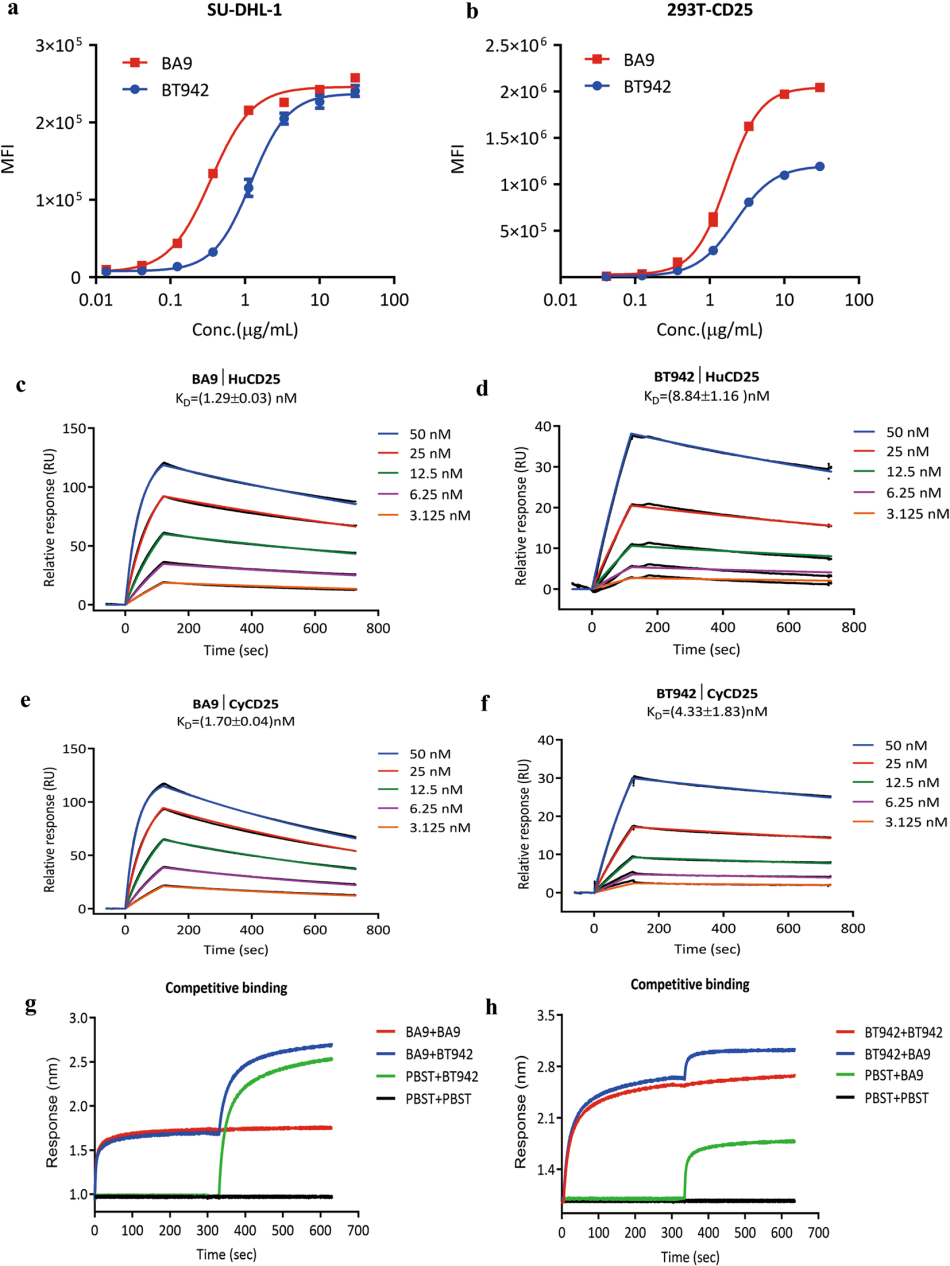
Binding activity to human or cynomolgus CD25 and direct competitive binding characteristics of BA9 and BT942. (**a**) BA9 and BT942 specifically bind to SU-DHL-1 cells determined by flow cytometry. Data were presented as Mean ± SEM. (**b**) BA9 and BT942 specifically bind to HEK293T-CD25 cells determined by flow cytometry. Data were presented as mean ± SEM. (**c**,**d**) Binding kinetics of BA9 (**c**) or BT942 (**d**) for Human CD25 were measured in a surface plasmon resonance (SPR) assay with BIAcore. Experiments were performed in triplicate. (**e**,**f**) Cross-reactivity with cynomolgus CD25 by BA9 (**e**) and BT942 (**f**) were measured in a surface plasmon resonance (SPR) assay with BIAcore. Experiments were performed in triplicate. (**g**,**h**) Direct competitive binding characteristics of BA9 and BT942 was performed using in-tandem format binning assay. SA sensors immobilized with biotinylated human CD25 were saturated with the first antibody or PBST, then exposed to the second antibody or PBST. Data of K_D_ were presented as mean ± SD.

Since it is difficult to obtain Treg cells from human tumors, we used Human diffuse tissue lymphoma cell line SU-DHL-1(CD25^+^) and human CD25 gene transfected HEK293T cells to perform in vitro cell-based evaluation with flow cytometry. Binding analysis of BA9 and BT942 revealed that both antibody candidates could specifically bind to SU-DHL-1 cells with EC50 of 0.35 μg/mL and 1.20 μg/mL, respectively (Fig. 2a). Similar results were seen using HEK293T-CD25 cell line with EC50 as 1.71 μg/mL and 2.21 μg/mL, respectively. BA9 also demonstrated higher top mean fluorescent intensity (Fig. 2b).

Binding kinetics of BT942 and BA9 to human CD25 were measured by surface plasmon resonance (SPR) using a Biacore 8K. The equilibrium constants (KD) of BA9 and BT942 with human CD25 were 1.29 ± 0.03 nM and 8.84 ± 1.16 nM, respectively (Fig. 2c,d). BA9 and BT942 also showed in the SPR assay they can react with cynomolgus CD25 with a KD of 1.70 ± 0.04 nM and 4.33 ± 1.83 nM, respectively (Fig. 2e,f). However, no reaction with mouse CD25 was observed (Supplementary Fig. S3). Notably, BA9 had higher binding affinity than BT942 in all the above in vitro data.

Given that BA9 and BT942 both do not prevent IL2 signaling, we measured the potential competitive binding of BA9 and BT942 to human CD25 using an Octet RED96 based epitope binding assay. For this biotinylated recombinant human CD25 protein was first immobilized on a streptavidin biosensor and then the biosensor was bound to saturation with excessive BA9 or BT942. Addition of BT942 to BA9 saturated sensor (Fig. 2g, blue) or BA9 to BT942 saturated sensor (Fig. 2h, blue) still showed binding response, indicating BA9 and BT942 do not block each other and bind distinct epitopes at human CD25.

### BT942 demonstrated weaker ADCC activity than BA9 in vitro

**Figure 3:**
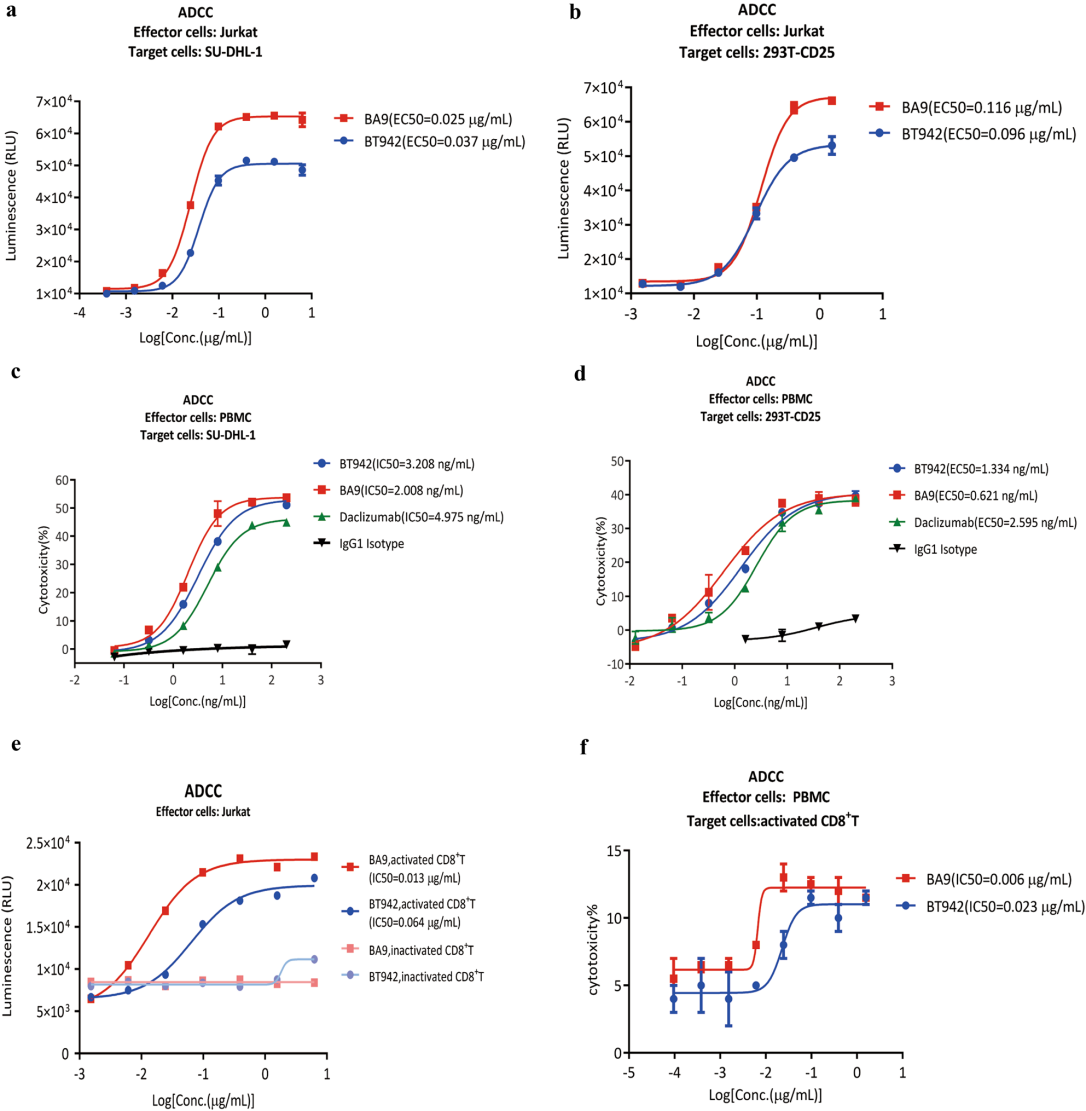
ADCC activity of BA9 and BT942. (**a**,**b**) ADCC was evaluated for BA9 and BT942 by a reporter bioassay. The target cells were SU-DHL-1 (**a**) or 293T-CD25 (**b**), the effector cells were Jurkat cells from Promega. BA9 can induce higher ADCC activity to both cell lines than BT942. Experiments were performed in duplicate. Data were presented as mean ± SEM. (**c**,**d**) ADCC was evaluated for BA9, BT942 and daclizumab by PBMC-mediated cytotoxicity. The target cells were SU-DHL-1 (**c**) or HEK293T-CD25 cells (**d**), the effector cells were PBMC. BA9 has higher ADCC activity than BT942. Experiments were performed in duplicate. Data were presented as mean ± SEM. (**e**,**f**) ADCC against activated CD8^+^T cells was evaluated for BA9 and BT942. The target cells were activated CD8^+^T cells. ADCC was evaluated by a reporter bioassay and the effector cells were Jurkat cells (**e**). ADCC was evaluated by PBMC-mediated cytotoxicity and the effector cells were PBMC (**f**). Experiments were performed in duplicate. Data were presented as mean ± SEM.

The antibody-dependent cell-mediated cytotoxicity (ADCC) is an important biological function attributed to the mechanism of action of Treg-deleting CD25 antibodies. The ADCC activity of BA9 and BT942 against SU-DHL-1 or HEK293T-CD25 were characterized using two assays. One is a reporter bioassay (Promega, G7940) with Jurkat cells as effector cells and luciferase substrate as reacting regent for monitoring Fc receptor signaling, the other is a cytotoxicity assay with PBMC as effector cells. BA9 and BT942 showed IC50 towards SU-DHL-1 as 0.025 μg/mL, 0.037 μg/mL, and IC50 towards HEK293T-CD25 as 0.116 μg/mL, 0.096 μg/mL, respectively, in the reporter bioassay. BA9 also demonstrated stronger top signaling (Fig. 3a,b). Furthermore, BA9, BT942 and daclizumab showed IC50 towards SU-DHL-1 as 2.008 ng/mL, 3.208 ng/mL, 4.975 ng/mL, and IC50 towards HEK293T-CD25 as 0.621 ng/mL, 1.334 ng/mL, 2.595 ng/mL respectively when evaluated by PBMC-mediated cytotoxicity (Fig. 3c,d). BT942 demonstrated weaker ADCC activity against SU-DHL-1 or HEK293T-CD25 cells with high CD25 expression. CD25 is also transiently expressed on activated T_eff_ cells although at a lower expression level than Treg cells. The unwanted ADCC activity of BT942 and BA9 against in vitro activated CD8^+^ T cells were also evaluated. BT942 showed weaker ADCC activity than BA9 on activated CD8^+^ T cells wheather evaluated by an ADCC reporter bioassay (Fig. 3e) or PBMC-mediated cytotoxicity (Fig. 3f).

### BA9 and BT942 demonstrated significant tumor suppression in early phase and late phase of tumor establishment

**Figure 4:**
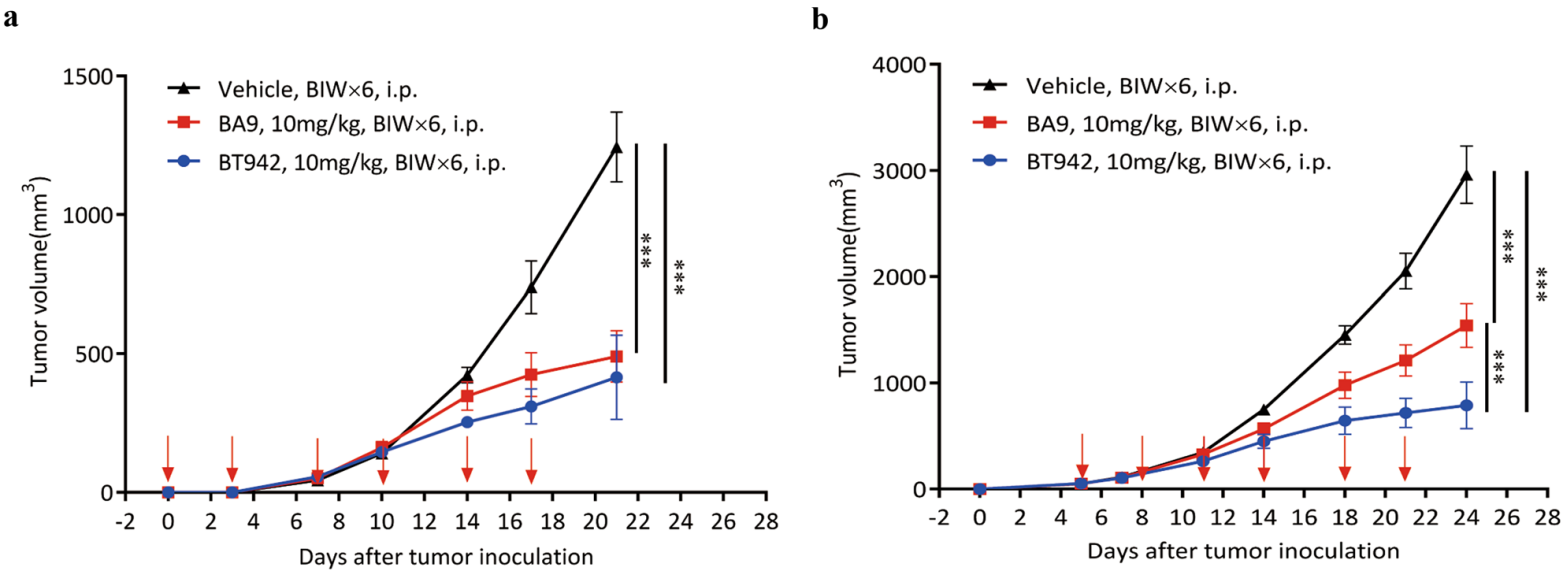
Antitumor effect of BA9 and BT942 in B-hIL2RA mice in MC38 tumor model for Early phase and Late phase treatment. (**a**) Early phase treatment: mean tumor volume of MC38 tumors in B-hIL2RA mice treated with vehicle versus anti-CD25 antibody (n = 8/group). Randomly, B-hIL2RA mice were grouped by weight and treated with anti-CD25 (10 mg/kg, i.p., twice a week). MC38 cells (5 × 10^5^ cells) were injected s.c. into mice in the next day (day 0). (**b**) Late phase treatment: mean tumor volume of MC38 tumors in B-hIL2RA mice treated with vehicle versus anti-CD25 (n = 8). MC38 cells (5 × 10^5^ cells) were injected subcutaneous into B-hIL2RA mice (day 0). Antibody treatments (10 mg/kg, i.p., twice a week) were started when tumors had grown to 50–60 mm^3^ (day 5). P values were calculated using two-way ANOVA (*p < 0.05, **p < 0.01, ***p < 0.001).

The effect of BA9 and BT942 on tumor suppression at early phase of tumor development was investigated in MC38 Model in B-hIL2RA humanized mice. B-hIL2RA mice are human CD25 humanized mice with CRISPR technology (human CD25+/+, mouse CD25−/−). Eight mice for each antibody were administered intraperitoneally at 10 mg/kg on day-1 (the day before tumor inoculation) with vehicle group as control. Tumor suppression ability of antibodies is monitored by tumor growth inhibition rate (TGI) which reflect a decrease percentage of the mean tumor volume for one treatment group compared with the vehicle group at one day. Both BA9 and BT942 significantly reduced tumor growth with the dose of 10 mg/kg twice a week (BIW); the tumor growth inhibition rate was 60.6% and 66.6% at day 21 after tumor inoculation, respectively (Fig. 4a).

To examine the effect of BA9 and BT942 on late phase tumor development 8 mice per group were administered at 10 mg/kg on day 5 when group mean tumor volumes had grown to 50–60 mm^3^. Like early phase results the antibodies demonstrated remarkable reduced tumor growth; the TGI for BA9 and BT942 was 48.7% and 74.7% at day 24 after tumor inoculation, respectively (Fig. 4b). Overall, both BA9 and BT942 showed significant tumor suppression effects whether administered in the early or late phase of tumor establishment.

**Figure 5:**
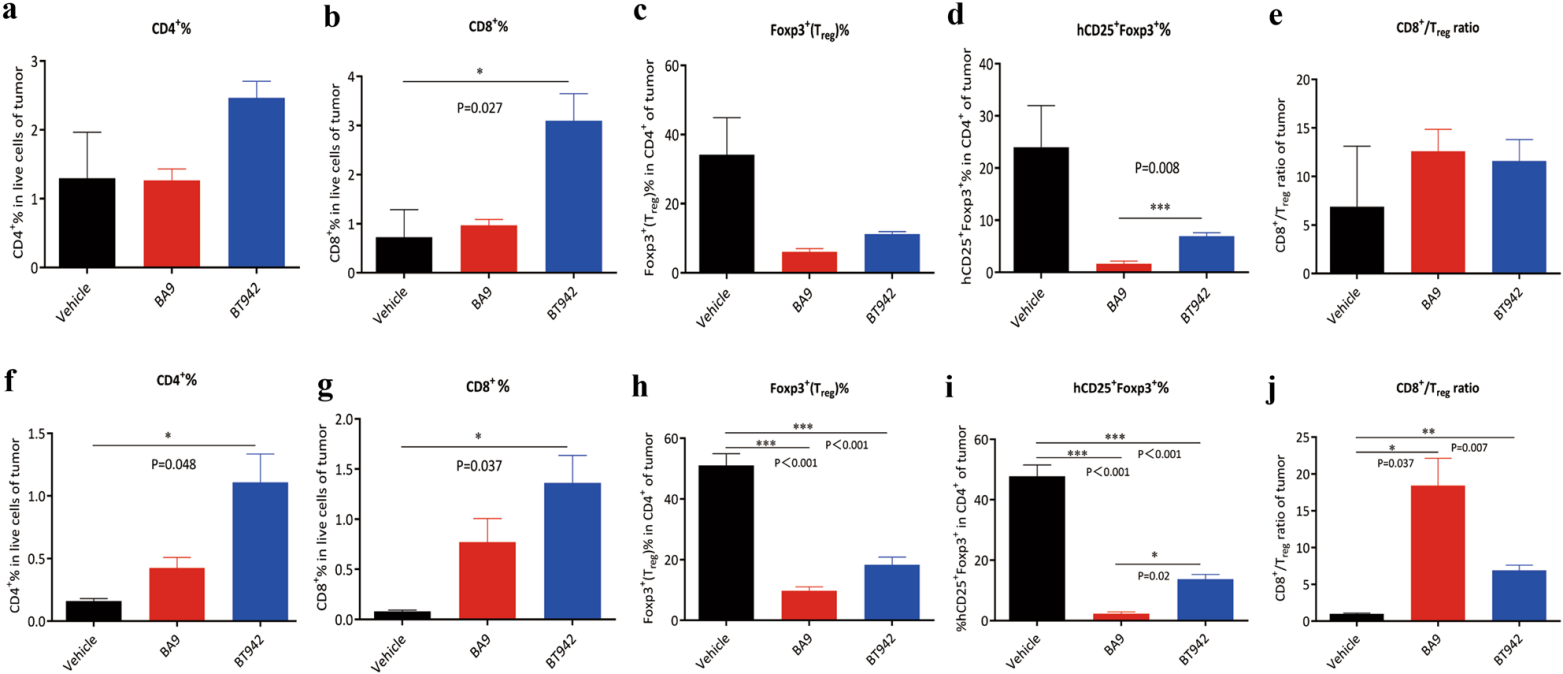
The immune cell population analysis after BA9 and BT942 treatment in MC38 tumor in B-hIL2RA mice. (**a**–**e**) Quantification of CD4^+^ T, CD8^+^ T, Foxp3^+^ (Treg) and hCD25^+^Foxp3^+^ population percentage in MC38 tumors from early phase treatment. CD8^+^/Treg cell ratios were also analyzed. p values obtained by one-way ANOVA. Data were presented as mean ± SEM. (**f**,**g**,**h**,**i**,**j**) Quantification of CD4^+^ T, CD8^+^ T, Foxp3^+^ (Treg) and hCD25^+^Foxp3^+^ population percentage in tumors from late phase treatment. CD8^+^/Treg cell ratios were also analyzed. p values obtained by one-way ANOVA. Data were presented as mean ± SEM. Bars without labels indicate that no statistical significance was observed.

The immune cell population in tumors and peripheral blood cells was also monitored in the same experiments. BT942 treatment resulted in higher increase in CD45^+^, CD3^+^T, CD4^+^ and CD8^+^T percentage in tumor at early or late phase treatment experiment when compared with vehicle or BA9 treatment. p-values of BT942 compared with vehicle were 0.027 for CD8^+^T at day 16 after tumor inoculation in early phase, 0.048 for CD4^+^T and 0.037 for CD8^+^T at day 24 after tumor inoculation in late phase, respectively (Fig. 5a,b,f,g). This difference was also seen in CD45^+^ (p-value = 0.001) and CD3^+^T (p-value = 0.001) proportions in tumor in late phase treatment (Supplementary Fig. S4). p-values of BT942 compared with BA9 were 0.013 for CD45^+^T and 0.029 for CD3^+^T in late phase treatment. This result is consistent with BT942’s weaker ADCC activity at activated CD8^+^ T cells in vitro. Both BA9 and BT942 reduced the proportion of hCD25^+^Foxp3^+^ cells and Foxp3^+^(Treg) cells in the tumor at early or late phase treatment experiment (Fig. 5c,d,h,i). Interestingly, there were more residual hCD25^+^Foxp3^+^ cells than BA9 (p-value = 0.008, 0.02) after BT942 treatment, in both early and late phase treatment, which is consistent with BT942’s weaker ADCC activity against high human CD25-expressing cells in vitro. While both BA9 and BT942 increased the CD8^+^/Treg cell ratios, BA9 treatment resulted in higher cell ratio as fewer residual Treg cells remain (Fig. 5e,j).

In the late phase treatment experiment, BA9 significantly reduced the proportion of hCD25^+^Foxp3^+^ cells and Treg cells in peripheral blood cells, but BT942 only had a slight effect (Supplementary Fig. S5). Unlike in tumor cells, there were no significant changes of CD45^+^, CD3^+^T, CD4^+^T, CD8^+^T proportion in peripheral blood in the late phase treatment experiment for both BA9 and BT942 (Supplementary Fig. S5).

### BT942 showed stronger activity to Treg and iTreg than activated CD4^+^ T and CD8^+^ T cells

**Figure 6:**
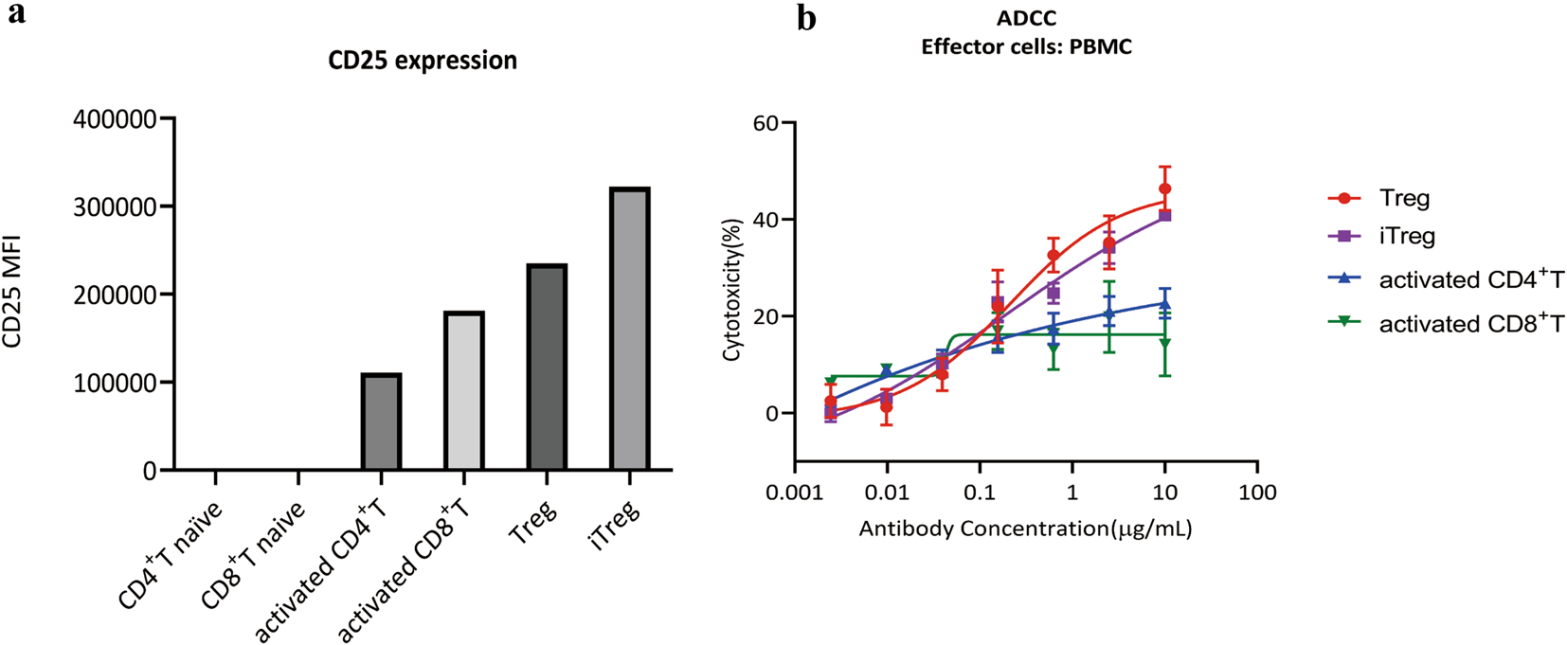
Comparison of CD25 expression level in human naïve CD4^+^ and CD8^+^ T, activated CD4^+^ and CD8^+^ T, Treg, iTreg cells and ADCC activity toward these different cell types by BT942. (**a**) Comparison of CD25 expression level in human naïve CD4^+^ and CD8^+^ T, activated CD4^+^ T and CD8^+^ T, Treg, iTreg cells. CD4^+^ and CD8^+^ T cells were activated by CD3/CD28 T Cell Activator for 48 h. CD4^+^, CD8^+^ naïve T cells and activated CD4^+^T, CD8^+^T cells were stained by anti-human CD4 or CD8 and CD25 fluorescent-labeled antibody. Treg and iTreg were stained by anti-human CD3, CD4, Foxp3 and CD25 fluorescent-labeled antibody. Cells were analyzed in flow cytometry by Beckman CytoFLEX. (**b**) ADCC activity toward these different cell types by BT942. The effector cell was PBMC.

As CD25 is transiently upregulated following T_eff_ cells activation, we examined cytotoxicity to activated CD4^+^ T and CD8^+^ T cells using an anti-CD25 antibody. Human CD25 expression levels in naïve CD4^+^ and CD8^+^ T, activated CD4^+^ and CD8^+^ T, Treg, iTreg cells were evaluated in Flow cytometry. iTreg was induced from CD4^+^T cells. Human CD25 expression levels of naïve CD4^+^ and CD8^+^ T cells were under detection limit. Treg and iTreg cells showed more CD25 expression than activated CD4^+^ T and CD8^+^ T cells (Fig. 6a). Furthermore, ADCC to these different cell types by BT942 was also evaluated (Fig. 6b). BT942 demonstrated stronger activity to Treg or iTreg than activated CD4^+^ T or CD8^+^ T cells.

### BT942 demonstrates improved efficacy than IL-2 blocking Daclizumab and synergizes with anti-PD1 to eradicate established tumors in vivo

**Figure 7:**
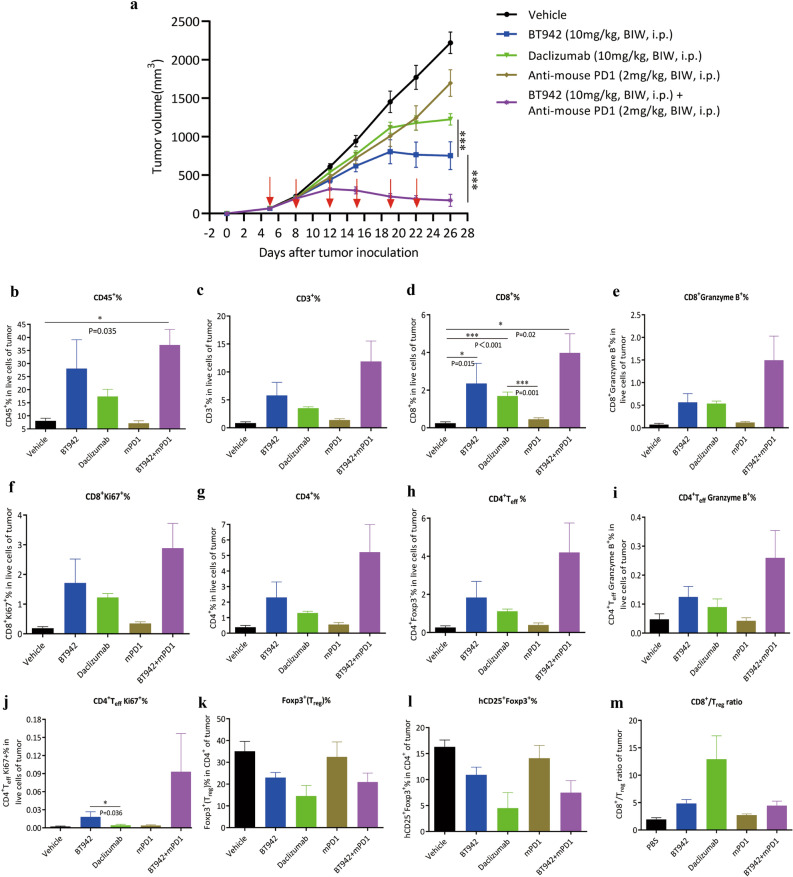
Comparison of in vivo antitumor effects of BT942 with daclizumab or combination of BT942 and anti-mouse PD1 antibody (n = 8). (**a**) Mean tumor growth volumes in different treatment groups. Mice were injected by BT942, Daclizumab (Sansheng Guojian), anti-mouse PD1, their combination of BT942 and anti-mouse PD1 antibody or vehicle (PBS) when mean tumor volumes reached 68 mm^3^ at day 5. Tumor size was measured twice a week by caliper. p values obtained by two-way ANOVA. (**b**–**l**) Quantification of CD45^+^, CD3^+^, CD8^+^, CD8^+^Granzyme B^+^, CD8^+^Ki67^+^, CD4^+^T, CD4^+^T_eff_ (CD4^+^FoxP3^−^), CD4 ^+^T_eff_ Granzyme B^+^ (CD4^+^FoxP3^−^Granzyme B^+^), CD4 ^+^T_eff_ Ki67^+^ (CD4^+^FoxP3^−^Ki67^+^), hCD25^+^Foxp3^+^ and Foxp3^+^ (Treg) cell population percentage in live cells in MC38 tumors. p values obtained by one-way ANOVA. Bars without labels indicate that no statistical significance was observed. (**m**) CD8^+^/Treg cell ratios were analyzed. p values obtained by one-way ANOVA. Bars without labels indicate that no statistical significance was observed. Data were presented as mean ± SEM.

To explore if the non-IL2 blocking properties improve its efficacy, we sought to compare the efficacy of BT942 with daclizumab which blocks IL-2 signalling on IL-2R in the MC38 syngeneic tumor model in B-hIL2RA humanized mice. MC38 cells (5 × 10^5^ cells) were injected subcutaneously into B-hIL2RA mice. Mice were randomized with 8 mice per group and treated with BT942, Daclizumab (Sunshine Guojian Pharmaceutical) or vehicle (PBS) when mean tumor volume reached 68 mm^3^ at day 5. Both BT942 and Daclizumab were dosed at 10 mg/kg intraperitoneally twice a week (BIW). Tumor growth inhibition rate (TGI) was used to define the antitumor efficacy of these mAbs. TGI of BT942 and daclizumab is 68.2% and 46.3% at day 26 after tumor inoculation, respectively. Non-IL2 blocking BT942 demonstrates significant stronger efficacy (Fig. 7a).

T_eff_ cell population in tumor is important for the efficacy of PD1 inhibitors. Treg's inhibition of T_eff_ cells in tumor is one of the reasons for the weak response of PD1/PD-L1 inhibitors. Whereas anti-CD25 antibody can delete the Treg and further increase the T_eff_ percentage in the tumor, we examined combining BT942 and anti-Mouse PD1(BioXcell, BE0146) to see if they had a synergistic effect in MC38 Model in the same experiment as above. Mice were treated with BT942, anti-mouse PD1, or their combination at 10 mg/kg intraperitoneally twice a week with vehicle group as control when tumors reached 68 mm^3^. The inhibitory rate of BT942 on the tumor is 68.2%, the inhibitory rate of BT942 and PD1 antibody on the tumor is 95.2%, the combined use significantly improved the effect of the single mAb (Fig. 7a, Supplementary Table S2). Individual data of tumor growth in each treatment group was showed in Supplementary Fig. S6.

Multiple immune cell populations were also analyzed in flow cytometry after BT942, daclizumab and combination treatment in the same experiments. Cells in tumor, Spleen and Lymph Nodes (inguinal, axillary) were collected at day 26 after tumor inoculation for flow cytometry analysis. BT942 treatment resulted in remarkable increase in CD45^+^, CD3^+^, CD8^+^, CD8^+^ granzyme B^+^, CD8^+^Ki67^+^, CD4^+^, CD4^+^T_eff_, CD4^+^T_eff_ granzyme B^+^ and CD4^+^T_eff_ Ki67^+^ T cell percentage in MC38 tumor (Fig. 7b–j). BT942 treatment resulted in a significant bigger increase in CD8^+^ T percentage when compared to vehicle treatment (p-value = 0.015) (Fig. 7d). Combination with BT942 and PD1 antibody resulted higher increase in these immune cell population percentage in the tumor, with p-values of 0.035 for CD45^+^and 0.02 for CD8^+^ T cells when compared to vehicle (Fig. 7b,d). BT942 treatment resulted in a slight bigger increase in these immune cell populations percentage than daclizumab except for CD8^+^ granzyme B^+^ T percentage. BT942, daclizumab and the combination all reduced the proportion of Foxp3^+^(Treg) and hCD25^+^Foxp3^+^ cells percentage in the tumor (Fig. 7k,l). While BT942, daclizumab and the combination all increased the CD8^+^/Treg cell ratios, daclizumab treatment resulted the highest cell ratio as its fewer residual Treg cells (Fig. 7m). Multiple immune cell populations percentage in Spleen and Lymph Nodes were also analyzed after treatment. The combination just significantly reduced the proportion of CD8^+^ population percentage in Spleen. Unlike in tumor, there was no significant changes of CD45^+^, CD3^+^, CD4^+^T and CD8^+^T proportion in Spleen and Lymph Nodes for both BT942 and their combination except for CD8^+^ population percentage in Spleen by combination (Supplementary Fig. S7). Both BT942 and the combination significantly reduced the proportion of hCD25^+^Foxp3^+^ cells and Treg cells in Spleen, but not in Lymph Nodes (Supplementary Fig. S7). CD25 levels of multiple immune cell populations from tumors and blood in vehicle group were also analyzed in flow cytometry at day 26 after tumor inoculation (Supplementary Fig. S8). CD4^+^Foxp3^+^ cells have the highest CD25 level than other cell populations.

### Pharmacokinetic and pharmacodynamic evaluation of BT942 in cynomolgus monkeys

**Figure 8:**
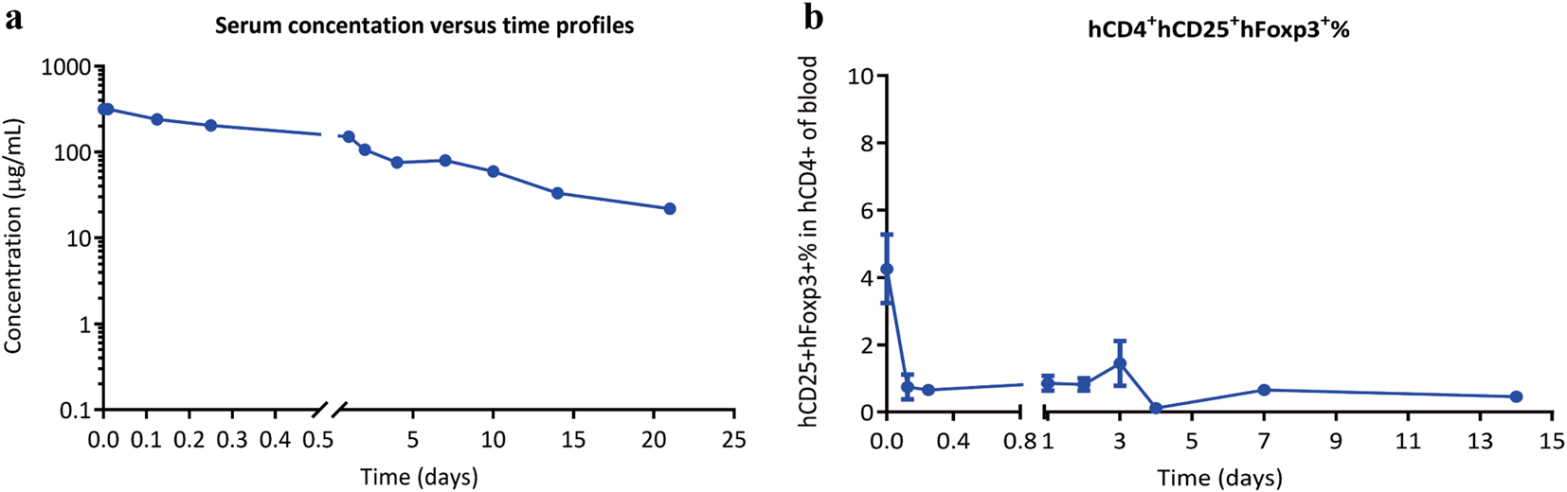
Pharmacokinetic and pharmacodynamic characteristics of BT942 in healthy cynomolgus monkeys. (**a**) Two healthy cynomolgus monkeys were administered intravenously at a single dose of 10 mg/kg with BT942. Enzyme-linked immunosorbent assay (ELISA) was used to determine the concentration of BT942 in serum. ELISA experiment was performed in triplicates. The main PK kinetic parameters were calculated using Winnolin software. Data were presented as mean ± SEM. (**b**) The peripheral blood before and after administration were collected from monkeys, and then the percentage of CD4^+^CD25^+^Foxp3^+^ cells in peripheral blood at different time points was analyzed with flow cytometry (CytomicsTM FC500). Data were presented as mean ± SEM.

As BT942 showed a significant efficacy in vivo we evaluated the pharmacokinetic (PK) and pharmacodynamic (PD) characteristics of this novel anti-CD25 antibody using healthy cynomolgus monkeys. For these experiments 2 animals were administered BT942 intravenously at doses of 10 mg/kg. ELISA was used to determine the concentration of BT942 in serum. Following a single-dose injection, BT942 showed a bi-exponential serum concentration–time profile with a short distribution phase followed by a long elimination phase, with a terminal half-life (t_1/2_, λz) of 206.97 ± 19.03 hours and AUC_(0−t)_ of 32,036.89 ± 1234.97 h*μg/mL (Fig. 8a; Supplementary Table S3). Interestingly, the pharmacodynamic tests showed that the percentage of CD4^+^CD25^+^Foxp3^+^ cells in peripheral blood decreased significantly a few hours after administration, and it was still very low until 14 days (Fig. 8b). This indicated that the effect of BT942 was durable. No signs of toxicity were observed in monkey examinations or mice treated with multiple doses of BT942.

### Structural analysis of the CD25-IL-2-BT942 complex

**Figure 9:**
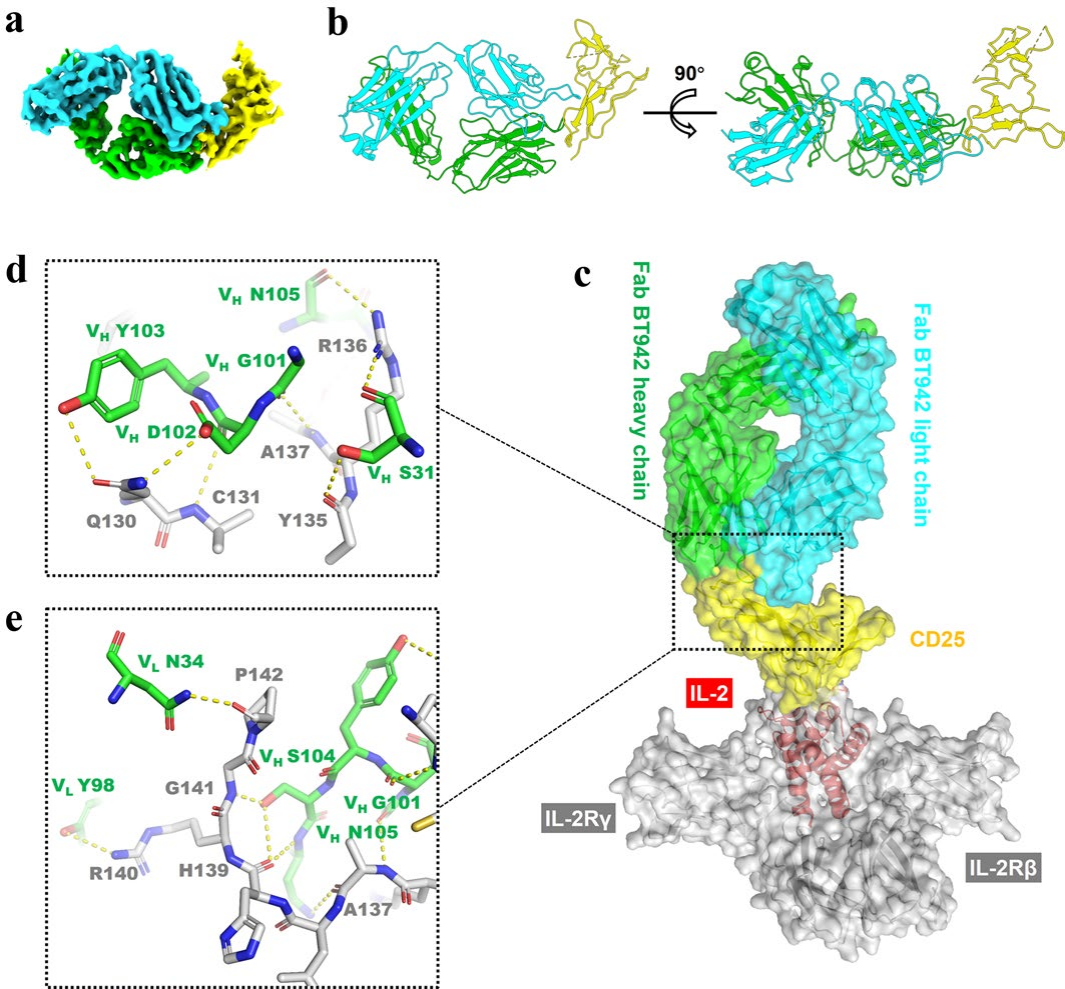
Cryo-EM structure of CD25-BT942 Fab complex. (**a**) The cryo-EM map of CD25-BT942 Fab complex. CD25 is colored in yellow, and the light chain and heavy chain of BT942 Fab is shown as cyan and green density, respectively. (**b**) The atomic model of CD25-BT942 Fab complex. Color codes are as in (**a**). The left and right panels show two different views of this complex. (**c**) BT942 Fab and IL-2 bind with CD25 in opposite direction according to the superposition of the CD25-BT942 Fab cryo-EM structure with the IL-2 quaternary signaling complex structure (PDB: 2B5I). The color codes of CD25-BT942 Fab complex are as in (**a**), with IL-2Rβ and IL-2Rγ shown as a grey transparent surface. IL-2 is shown in red to highlight its orientation. The boxed area represents the interface shown in (**d**) and (**e**). (**d**,**e**) The close-up views of the interface between CD25 and BT942 Fab showing the epitopes on CD25 (grey labels) and paratopes on BT942 (green labels). The hydrogen bonds are depicted as yellow dashed lines.

To investigate the interaction between BT942 and human CD25 protein we attempted to determine the structure of this complex using cryo-EM. However, the intrinsic flexibility of human CD25 protein had hindered our efforts to reconstruct the whole complex. Although CD25 was present in the complex, we could only get map of BT942 Fab with some extra blurred densities. To address this issue, we reconstituted the CD25-IL-2-BT942 Fab ternary complex for cryo-EM sample preparation. Raw cryo-EM micrographs and typical 2D class averages of this complex were shown in Supplementary Fig. S9. Fortunately, the binding of IL-2 stabilized CD25 further and we succeeded to get the density map of whole CD25-BT942 Fab complex. Density of IL-2 could be seen only if the threshold of the map was set to an extremely low value, suggesting the remaining flexibility of CD25. Considering the poor quality of IL-2 density in the cryo-EM reconstruction, we described the structure of CD25-BT942 Fab complex omitting IL-2 (Fig. 9a). Details of sample preparation, data collection and data processing are reported in the methods and Supplementary Table S4. The resolution of the final reconstruction reached 3.2 Å, which allows us to analyze the details of interactions between CD25 and BT942 (Fig. 9b). In this structure the binding site of BT942 at CD25 is on the opposite side of the CD25-IL-2 binding site (Fig. 9c), consistent with the observations in vitro where no IL-2 signaling blocking was observed.

Hydrogen bonds and salt bridges around CD25 residues Q130, C131, Y135, R136, A137, H139, R140, G141 and P142 contributed to the interaction between CD25 and BT942 (Fig. 9d,e). S31 from BT942 heavy chain CDR1, G101, D102, Y103, N105, S104 from CDR3, N34 from light chain CDR1, Y98 from light chain CDR3 contributed to this interaction (Fig. 9d,e).

## Discussion

Tregs protect cancer cells from immune attack. Tregs express CD25 on their surface and can be targeted for killing by antibodies and immunoconjugates targeting CD25. These antibodies have not been effective in controlling tumor growth of patients, probably because they also kill cytotoxic CD8^+^ T cells expressing CD25 that are needed for antitumor activity. Here we describe two anti-CD25 monoclonal antibodies, BT942 and BA9, that were obtained through immunizing human antibody transgenic mice with recombinant human CD25 protein followed by phage display for library construction and potential hits screening with human CD25 protein. BT942 has significant efficacy on both early and late stage MC38 colon cancer models, whether used alone or in combination with anti-PD1. The previously examined anti-CD25 monoclonal antibodies are either effective only in the early stages of tumors^20^, or only have a significant efficacy in combination with anti-PD1^26^.

BT942 has weaker binding activity to human CD25 and weaker ADCC activity against human CD25-expressing cells compared with BA9. Nevertheless, BT942 also demonstrated significant efficacy and led to a higher CD45^+^, CD8^+^ T and CD4^+^ T cell increase than BA9 in MC38 model. CD25 is also transiently expressed on activated T_eff_ cells although at lower percentage of the population and a lower expression level than Treg cells. BT942 showed a weaker ADCC activity than BA9 against in vitro activated CD8^+^ T cells wheather evaluated by an ADCC reporter bioassay or by PBMC-mediated cytotoxicity. A clear limitation of the study is the difficulties around conducting this type of in vivo work, here the sample size was small with one group of 8 mice tested. Additionally, only a fixed duration of 21–24 days following tumor inoculation was examined. Further experimentation by the field would be required to demonstrate clear differences between BA9 and BT942 and strengthen the outcomes seen in this study. The anti-tumor efficacy by BT942 in the B16-F10, Lewis lung carcinoma (LLC) and MCA205 Mouse Fibrosarcoma model was also explored but it did not demonstrate significant efficacy as single agent in these models.

Non-IL2 blocking BT942 also demonstrates significant stronger efficacy than the IL-2 blocking daclizumab in the MC38 syngeneic tumor model. This suggest non-IL2 blocking property is a necessary feature for the efficacy of anti-CD25 antibodies.

In addition, tumor growth inhibition of BT942 demonstrated synergy in combination with PD1 antibody using MC38 model and is higher than that of BT942 or anti-mouse PD1 alone. In terms of toxicity, the removal of Treg cells could trigger the excessive immune responses to microbial antigens, which will lead to the T cells hyper-reaction to intestinal commensal bacteria, causing inflammatory bowel disease (IBD), and other diseases^31^. For BT942, there was no significant reduction in animal weight and clinical observation did not find any abnormalities in mice efficacy study or in monkey PD\PK study.

No CD25 antibodies have demonstrated efficacy against cancer in clinic. BT942 appears to be an excellent candidate for immunotherapy of cancer.

## Methods

### Mice, cell lines and reagents

Reagents, cell lines and viral strains used in this study are listed in Supplementary Table 1.

### Generation of anti-CD25 antibodies

The mice used for immunization are human antibody transgenic mice that generated by our company. They were bred and kept under specific-pathogen free conditions. All animal experiments were complied with relevant ethical regulations regarding animal research. Protocols of mice experiments for immunization were approved by LUYE PHARMA Animal Experimentation and Ethics Committee. Human antibody transgenic mice were sequentially immunized with human CD25. In the meantime, titers of antibodies in the mice serum were tested by ELISA. Three days after the last immunization, the spleen was harvested for library construction. The construct of the phage library was carried out according to the method described in *Phage Display: A laboratory manual*^32^. Plates coated with human CD25 or streptavidin-magnetic beads binding biotin-CD25 were used to capture interest phages. Enriched phages were used to infect *E. coli* TG1 for expressing single-chain variable fragments (scFvs) and the binding and blocking activity were tested by ELISA. Positive clones were obtained and sequenced.

### ELISA-based binding assay for scFvs

Human CD25 protein (10165-H08H, Sino Biological) was coated on high binding ELISA plates with 0.2 μg/mL at 4 °C overnight, and then the plates were blocked with 3% skim milk powder in PBST (PBS containing 0.05% Tween-20) at 37 °C for 1 h. After washing two times with PBST, 100 μL Scfvs of different clones were added to each well, incubated at 37 °C for 1 h. Plates were washed two times and then HRP anti-M13 mAb was used to detect Scfv binding to human CD25.

### ELISA-based blocking assay for scFvs

Human CD25 protein (10165-H08H, Sino Biological) was coated on high binding ELISA plates with 0.5 μg/mL at 4 °C overnight. Plates were blocked with 3% skim milk powder in PBST (PBS containing 0.05% Tween-20) at 37 °C for 1 h. The mixture of IL2-biotin (0.03 μg/mL) and scFvs were added to the blocked ELISA plate and incubated at 37 °C for 1 h. After washing, the biotinylated IL2 binding to coated CD25 was detected by HRP-conjugated Streptomycin.

### Antibodies production

Heavy chain variable region and light chain variable region were amplified (2 × Phanta Max Master Mix, Vazyme, P515-01) using the positive clones screened from the library as templates. Overlap PCR was conducted to assemble variable region and signal peptide. Purified gene fragments were separately fused (ClonExpress II One Step Cloning Kit, Vazyme, C112-01) into the linearized pcDNA3.4 vectors with human IgG1 constant regions. The recombinant plasmids were prepared for production. Antibodies were expressed with Expi-CHO Expression system (A29133, Gibco) for 12 days and the supernatants were harvested and purified by a AT Protein A Diamond (AA0272, Bestchrom).

### ELISA-based binding assay

Human CD25 protein (10165-H08H, Sino Biological) was coated on high binding ELISA plates with 0.2 μg/mL at 4 °C overnight, and then the plates were blocked with 3% skim milk powder in PBST (PBS containing 0.05% Tween-20) at 37 °C for 1 h. After washing two times with PBST, serially diluted antibodies were added to each well, incubated at 37 °C for 1 h. Plates were washed two times and then HRP-goat anti-human IgG mAb (474-1006, KPL) was used to detect antibodies binding to CD25. Experiments were performed in triplicate, value = mean ± SEM.

### Cell based binding assay

SU-DHL-1(CRL-2955, ATCC) or HEK293T-CD25 cells were harvested and washed by FACS buffer (0.2% BSA in PBS) two times. Serially diluted antibodies were mixed with 1 × 10^6^ cells at 4 °C for 1 h. After washing two times by FACS buffer, cells were incubated in dark with Goat Anti-Human IgG-PE (2040-09, Southern Biotech) at 4 °C for 30 min and then analyzed by NovoCyte 2060R flow cytometry. Experiments were performed in triplicate, value = mean ± SEM.

### ADCC reporter bioassay

ADCC reporter bioassay was conducted with SU-DHL-1 cells, HEK293T-CD25 cells or CD8^+^ T cells as target cells and Jurkat cells (G7011, Promega) as effector cells. Activated CD8^+^ T cells were got by stimulating CD8^+^ T cells for 72 h with OKT3, CD28 antibody and IL-2. 25 μL target cells at 1.2 E6/mL, serially diluted antibodies and Jurkat cells at 2.4 E6/mL were sequentially added to a White Tissue Culture treated 96 well plate in a cell incubator for 5 h. Bio-Glo Luciferase Assay Buffer/Substrate (G7940, Promega) was added to react for 15 min and then the value of Luminescence was read on Tecan microplate reader. Experiments were performed in duplicate, value = mean ± SEM.

### In vitro ADCC assay

Antibody-dependent cell-mediated cytotoxicity was conducted with SU-DHL-1 cells, inactivated CD8^+^ T (PB009-3-C, ALLCELLS) cells or activated CD8^+^ T cells as target cells and PBMCs as effector cells. Activated CD8^+^ T cells were got by stimulating CD8^+^ T cells for 72 h with OKT3, CD28 antibody and IL-2. Serially diluted antibodies were mixed with target cells in 96-well plates at 37 °C for 15–30 min. The effector cells PBMCs (PB003F-C, ALLCELLS) were added and the mixture was incubated at 37 °C for 5 h. After substrate was added, OD490 value was read on the microplate reader. Percent cytotoxicity was computed using following formula: 100 × (OD490 of Experimental wells–OD490 of Experimental Wells Without Antibody)/(OD490 of Target Cells Maximum LDH Release wells-OD490 of Target Cells Spontaneous LDH Release wells). Experiments were performed in duplicate, value = mean ± SEM.

### Affinity analysis by surface plasmon resonance

SPR measurements were performed at room temperature using a BIAcore 8K system with CM5 chip, which was amino coupled by human antibody capture kit. HBS-EP + buffer (150 mM NaCl, 10 mM HEPES, 3 mM EDTA and 0.05% (v/v) surfactant P20 pH 7.4) was used as running buffer. The blank channel of the chip served as the negative control. The antibodies were captured on the chip at 400–500 response units. Serial dilutions of human or cynomolgus CD25 (from 50 to 3.125 nM with twofold dilution) were applied to flow over the chip surface, which was regenerated with 3 M MgCl_2_ after each cycle. The affinity was calculated using a 1:1 (Langmuir) binding fit model or two state reaction model with BIA evaluation software. Experiments were performed in duplicate, value = mean ± SD.

### ELISA-based IL2-binding inhibition assay

Human CD25 protein (10165-H08H, Sino Biological) was coated on high binding ELISA plates with 0.5 μg/mL at 4 °C overnight. Plates were blocked with 3% skim milk powder in PBST (PBS containing 0.05% Tween-20) at 37 °C for 1 h. The mixture of IL2-biotin (0.03 μg/mL) and serially diluted CD25 antibodies were added to the blocked ELISA plate and incubated at 37 °C for 1 h. After washing, the biotinylated IL2 binding to coated CD25 was detected by HRP-conjugated Streptomycin. Experiments were performed in duplicate, value = mean ± SEM.

### In vitro IL-2 signaling by STAT5 phosphorylation assay

PBMCs were co-cultured with 10 μg/mL CD25 antibody in a 96-U bottom plate for 30 min, then 10 U/mL IL2 was added and cultured for 10 min (working medium: 1640 + 10% FBS, containing 2 mM l-Glutamine). Cell suspension was prepared as follows. 200 µL Foxp3 fixation/breaking membrane working solution was added to cell pellet in each well and incubated for 30–60 min at 2–8 °C or room temperature in the dark. After the sample was centrifuged at 400–600*g* for 5 min at room temperature, the supernatant was discarded and plates were washed twice with 200 µL/well 1× rupture solution and once with PBS at room temperature. Ice-cold Phosflow™ Perm Buffer III was slowly added and then incubated on ice for 30 min. After washing two times by FACS buffer, cells were stained with fluorescent-labeled antibody at 4 °C for 30 min and then analyzed by Beckman CytoFLEX flow cytometry. Experiments were performed in triplicate, value = mean ± SEM.

### In vitro IL-2 signaling by T cell activation assay

Human CD3^+^ T cells were activated by CD3/CD28 T Cell Activator (StemCell) for 48 h in the presence of 10 μg/mL CD25 antibody in 1640 + 10% FBS (Gibco) medium. After staining by Fixable Viability Kit (Biolegend, 423105) for 15 min, cells were washed by two times. Cells were then stained by mixture of anti-human CD3, CD4 and CD8 fluorescent-labeled antibody at 4 °C for 20 min and washed. After resuspending by 1 × Fixation/Permeabilization buffer, cells were incubated in dark for 30 min at room temperature and then resuspended by 1 × Permeabilization buffer. After centrifugation, cells were stained by Ki67 and Granzyme B fluorescent-labeled antibody for 30 min and then analyzed by Beckman CytoFLEX flow cytometry. Experiments were performed in triplicate, value = mean ± SEM.

### CD25 expression level in naïve CD4^+^ and CD8^+^ T, activated CD4^+^ T and CD8^+^ T cells, Treg, iTreg and ADCC activity toward to these different cell types by BT942

Treg, CD4^+^ T cells and CD8^+^ T cells were isolated from PBMC. CD4^+^ T and CD8^+^ T cells were activated by CD3/CD28 T Cell Activator (StemCell) for 48 h.

In vitro induced Treg (iTreg) were induced from CD4^+^ T cells. CD4^+^ T cells were activated by CD3/CD28 T Cell Activator for 7 days in X-Vivo 15 medium containing 10% FBS, 1% Glutamax, 2 mg/mL *N*-acetylcysteine, 1 × sodium pyruvate, 1 × HEPES, 1 × nonessential amino acids, 1 × pen/strep, 50 μM 2-mercaptoethanol, 500 U/mL IL2, 100 ng/mL rapamycin and 10 ng/mL TGFβ1. For CD25 expression assay, CD4^+^, CD8^+^ naïve T cells and activated CD4^+^T, CD8^+^T cells were stained by anti-human CD4 or CD8 and CD25 fluorescent-labeled antibody. Treg and iTreg were stained by anti-human CD3, CD4, Foxp3 and CD25 fluorescent-labeled antibody. Cells were analyzed by Beckman CytoFLEX flow cytometry.

Methods of ADCC assay for these cell types was the same as described in “In vitro ADCC assay”.

### Direct competitive binding characteristics of BA9 and BT942

Direct competitive binding of the antibodies was performed on a ForteBio Octet Red96 system (ForteBio) using in-tandem format binning assay. Biotinylated CD25 was loaded onto SA sensors (18-5019, fortebio). The sensors were then exposed to the first antibody at 50 µg/mL or PBST for 300 s, then to the second antibody at 50 µg/mL or PBST for 300 s. Data was processed using ForteBio’s Data Analysis Software 9.0.

### Syngeneic mouse models

All animal works were carried out in compliance with ARRIVE guidelines (https://arriveguidelines.org). These experiments were carried out at Beijing Biocytogen Co. Ltd. B-hIL2RA humanized mice (C57BL/6-Cd25^tm1(CD25)^/Bcgen) were used for these efficacy studies.

For early phase of tumor development, mice were randomized into three groups, with 8 mice per group based on their body weight at day-1 (the day before tumor inoculation). Then BA9 and BT942 were dosed at 10 mg/kg intraperitoneally twice a week with vehicle group as the control. 5 × 10^5^ MC38 cells in PBS were inoculated subcutaneously in the flank of mice at the next day (day 0). At day 16 after tumor inoculation, three mice in each group were sacrificed to test the proportion of CD45^+^, CD3^+^T, CD4^+^T, CD8^+^T, CD25^+^Foxp3^+^, Foxp3^+^(Treg) cells in tumor by flow cytometry. Remaining animals were euthanized by CO_2_ asphyxiation when the mean tumor volume reached about 1300 mm^3^ at day 21 after tumor inoculation.

For late phase of tumor development, B-hIL2RA humanized mice were implanted with 5 × 10^5^ cells MC38 cells subcutaneously in the flank. Mice were distributed into three groups (n = 8) with group mean starting volumes of 50 to 60 mm^3^ (day 5). Then BA9 and BT942 were also dosed at 10 mg/kg intraperitoneally twice a week with vehicle group as the control. Animals were euthanized by CO_2_ asphyxiation when the mean tumor volume reached about 2000 mm^3^ at day 24 after tumor inoculation. Four mice with mean tumor volume close to the whole group were selected to test the proportion of CD45^+^, CD3^+^T, CD4^+^T, CD8^+^T, CD25^+^Foxp3^+^, Foxp3^+^(Treg) cells in tumor and peripheral blood by flow cytometry.

For the synergistic effect of BT942 and anti-mouse PD1 antibody at late phase of tumor development, B-hIL2RA humanized mice were implanted with 5 × 10^5^ MC38 cells subcutaneously in the flank. Mice were distributed into five groups with 8 mice per group and treated with BT942, Daclizumab (Sunshine Guojian Pharmaceutical) which block IL-2 signaling, anti-mouse PD1 (BioXCell), their combination (BT942 combined with the anti-mouse PD1) or vehicle (PBS) when mean tumor volume reached 68 mm3 (day 5). Animals were euthanized by CO_2_ asphyxiation when mean tumor volume reached about 2000 mm^3^ at day 26 after tumor inoculation. Four mice with mean tumor volume close to the whole group were selected to test the proportion of CD45^+^, CD3^+^T, CD4^+^T, activated CD4^+^T_eff_, living CD4^+^T_eff_, Foxp3^+^(Treg), CD25^+^Foxp3^+^, CD8^+^T, living CD8^+^T, activated CD8^+^T, CD8^+^Granzyme B^+^CD25^+^ cells in tumor and peripheral blood by flow cytometry. And we also tested the proportion of CD45^+^, CD3^+^T, CD4^+^T, CD8^+^T, Foxp3^+^(Treg), CD25^+^Foxp3^+^ cells in spleen and lymph node by flow cytometry.

Tumor size was measured twice a week by caliper. Tumor volumes were calculated as volume = (w^2^ × l)/2 where w is the tumor width and l is the tumor length in millimeters.

The Tumor Growth Inhibition rate (TGI) were calculated as TGI (%) = [1 − (Ti − T0)/(Vi − V0)] × 100%. (Ti: Mean tumor volume of the treatment group at day i after tumor inoculation, T0: Mean tumor volume of the treatment group at the day of first administration; Vi: Mean tumor volume of the vehicle group at day i after tumor inoculation, V0: Mean tumor volume of the vehicle group at the day of first administration).

### Flow cytometry analysis for multiple immune cell populations after treatment in B-hIL2RA mice

For flow cytometry experiments, blood and tissues were collected and processed after animals were euthanized. Spleen, LNs (inguinal, axillary) and tumors were dissected into RPMI1640.

Aspiration of each blood sample 100 μL was transferred into a 1.5 mL EP tube. Add 1.4 mL of red blood cell lysate, Turn upside down mix, and lyse for 5 min at room temperature. Add 10 mL of PBS to stop the lysis.

Transfer the spleen to a 35 mm culture dish, and then grind with a sterile syringe tail. Wash and centrifuge at 500*g* for 5 min at 4 °C. Resuspend the cell pellets with 2 mL of red blood cell lysis buffer and incubate for 5–10 min at room temperature. Add 10 mL of PBS to stop the lysis.

Transfer the lymph node to a 35 mm culture dish, and then grind with a sterile syringe tail. Wash and centrifuge at 500*g* for 5 min at 4 °C. Resuspend cells in PBS to the required volume for further applications.

Cut the tumor into small pieces of 2–4 mm, Transfer the tissue into the tube containing the enzyme mix (Tumor Dissociation Kit, Miltenyi Biotec) and placed on the gentleMACS Octo Dissociator, Performing semi-automatic procedures and then grind with a sterile syringe tail with 10 mL of RPMI 1640 at 4 °C.

All cells (blood, tumor, spleen and LNs) were dispersed through a 70 μm filter and centrifuge at 500*g* for 5 min at 4 °C. Count cells using a blood cell counting chamber. Resuspend cells in PBS to the required volume for further applications.

Samples were stained with Fixable Viability Dye eFluor™506 and anti-mouse CD45, CD3, CD4, CD8, anti-human CD25. Intracellular staining of FoxP3, Granzyme B and Ki67 was performed using the FoxP3 Transcription Factor Staining Buffer Set. All antibodies for flow cytometry are listed in Supplementary Table 1.

After incubation, CD45^+^, CD3^+^T, CD4^+^T, activated CD4^+^T_eff_ (CD4^+^FoxP3^−^), living CD4 ^+^T_eff_ (CD4^+^FoxP3^−^Granzyme B^+^ or CD4^+^FoxP3^−^Ki67^+^), CD8^+^T, living CD8^+^T (CD8^+^Granzyme B^+^ or CD8^+^Ki67^+^) in live cells and Treg (CD4^+^FoxP3^+^), hCD25^+^Foxp3^+^ in CD4^+^T cells, and activated CD8^+^T (CD8^+^CD25^+^), CD8^+^Granzyme B^+^CD25^+^ in CD8^+^T cells were gated and analyzed by Attune NxT Flow Cytometer (Thermo Fisher).

### Pharmacokinetics and pharmacodynamics experiments

All animal care and experimental procedures were complied with relevant ethical regulations regarding animal research. Pharmacokinetics and pharmacodynamic study protocols in monkeys were approved by Institutional Animal Care and Use Committee (IACUC) and the Approval Number was UPP-IACUC-2020-00000. The BT942 antibody was administered intravenously to cynomolgus monkeys (N = 2, 2 males, body weight 3 ~ 5 kg) at a dose of 10 mg/kg. Peripheral bloods were collected at predose and 1 min, 30 min, 1 h, 3 h, 6 h, 24 h, 48 h, 96 h, 168 h, 240 h, 336 h, 504 h post-dose for PK study. ELISA was used to determine the concentration of BT942 in serum. In this method, CD25 protein was used as the capture reagent, and goat anti-human IgG, monkey ads-HRP was detecting agent. Results are shown as mean ± SEM. The main PK kinetic parameters were calculated using Phoenix WinNonlin.

Peripheral bloods were collected at pre-dose and 1 min, 3 h, 6 h, 24 h, 48 h, 72 h, 168 h, 336 h post-dose to determine CD4^+^CD25^+^FoxP3^+^ percentage in CD4 + T cells with flow cytometry (CytomicsTM FC500).

### Cryo-electron microscopy analysis of the CD25-IL-2-Fab complex

#### Sample preparation

27 μL CD25 (extra cellular domain) at the concentration of 1.3 mg/mL was incubated with 20 μL IL-2 (1.76 mg/mL) and 30 μL BT942 Fab (2.5 mg/mL) on ice for 30 min and then the mixture was loaded onto a Superdex 200 SEC column. The eluted peak was applied to prepare cryo-EM grids. Frozen-hydrated cryo-EM specimens were prepared with a Thermo Fisher Vitrobot Mark IV plunger. 4 μL of CD25-IL-2-Fab complex was placed on a glow discharged holey carbon grid (Quantifoil Au R1.2/1.3) coated with thin layer of graphene oxide. The excess of solution on the grid was blotted for 1.0 s at 100% humidity at 8 °C before the grid was flash frozen in liquid ethane slush cooled at liquid-nitrogen temperature.

#### Data collection

Cryo-EM data were collected on a Thermo Fisher Titan Krios G3i electron microscope equipped with a Gatan K3 direct electron counting camera. The microscope was operated at 300 kV, and images of the specimen were recorded with a defocus range of − 1.4 to − 2.4 μm at a calibrated magnification of 130 k in super-resolution mode of the K3 camera, thus yielding a pixel size of 0.27 Å on the object scale. A total of 5836 movie stacks, each containing 32 sub-frames, were recorded with the semi-automated low-dose acquisition program EPU, with a total accumulated dose of 50 electrons/Å^2^.

#### Data processing

The raw super-resolution dose-fractionated images stacks were 2 × Fourier binned, aligned, dose-weighted and summed using MotionCor2, resulting in summed micrographs in a pixel size of 0.54 Å. Contrast transfer function (CTF) parameters were estimated using CTFFIND4.1. The following processing steps were performed in RELION3.1. First Laplacian-of-Gaussian method was used to pick particles automatically. Then all these particles were subjected to several rounds of reference-free 2D classification to remove contaminants and bad particles. After that 3D classification was performed using a map derived from PDB model as the initial reference model. The most homogeneous particles were selected for the final 3D auto-refinement. Reconstruction resolutions were determined based on the gold-standard Fourier shell correlation (FSC) 0.143 criterion with the high-resolution noise substitution.

### Data analysis

All one-way ANOVA statistical analyses were performed in SPSS Statistics 21 Software. All two-way ANOVA statistical analyses were performed in GraphPad Prism 8 Software. Details on the statistical tests applied are provided within the figure legends. The data are reported as bar graphs displaying individual values and means ± SEM, as indicated in the figure legends. No experiments were excluded from the analyses. p values were calculated using one-way ANOVA or two-way ANOVA (*p < 0.05, **p < 0.01, ***p < 0.001). EC50 values were performed in GraphPad Prism 8 Software using the inbuilt nonlinear regression curve fit (log (agonist) versus response, variable slope, 4 parameter).

## Supplementary Information

Supplementary Information.

## Data Availability

The variable region sequences of BT942 MAb have been deposited in GenBank with the accession codes MW251337 for heavy chain and MW251338 for light chain. The variable region sequences of BA9 MAb have been deposited in GenBank with the accession codes MW251339 for heavy chain and MW251340 for light chain. The structure of CD25 in complex with Fab has been deposited in the Protein Data Bank, with PDB code 7F9W. The corresponding cryo-EM map is available in the Electron Microscopy Data Bank, with code EMD-31499.
