# Supplementary material for: Two novel human anti-CD25 antibodies with antitumor activity inversely related to their affinity and in vitro activity
Source: Sci Rep. 2021 Nov 25;11:22966. doi: 10.1038/s41598-021-02449-y (PMC8617198; doi:10.1038/s41598-021-02449-y)
Supplement: Supplementary file 1 — Supplementary Information. [file 41598_2021_2449_MOESM1_ESM.docx]

Supplementary Materials for
**Two novel human anti-CD25 antibodies with antitumor activity inversely related to their affinity and *in vitro* activity**

Deyong Song^1,+^, Xiu Liu^1,+^, Chuangchuang Dong^1^, Qiaoping Wang^1^, Chunjie Sha^2^, Chuan Liu^3^, Zhenfei Ning^1^, Jing Han^1^, Hong Liu^1^, Mengqi Zong^1^, Yanyan Zhao^1^, Ying Li^1^, Guangsheng Liu^1^, Xin Shao^1^, Changlin Dou^1,*^

^+^These authors contributed equally to this work.

Corresponding author:

^*^Corresponding author. e-mail: DouChanglin@boan-bio.com (C.D.)

**This PDF file includes:**
Supplementary Fig. 1 to Fig. 9
Supplementary Table 1 to 4

**phage** display

**screening**

**sequencing**

VH

CH

VL

CL

**Antibody**

**expression**


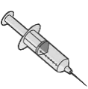

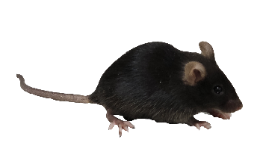


**In vitro** assay

**In vivo** assay

**Human antibody**

**transgenic mice**

**Supplementary Figure S1.** The candidate antibodies were obtained by phage display screening. There were 14 clones that do not block IL-2 binding to CD25 in 134 binding positive clones. Combined with other in vitro data such as ADCC and FACS, we have identified candidate antibodies for *in vivo* experiments.

**
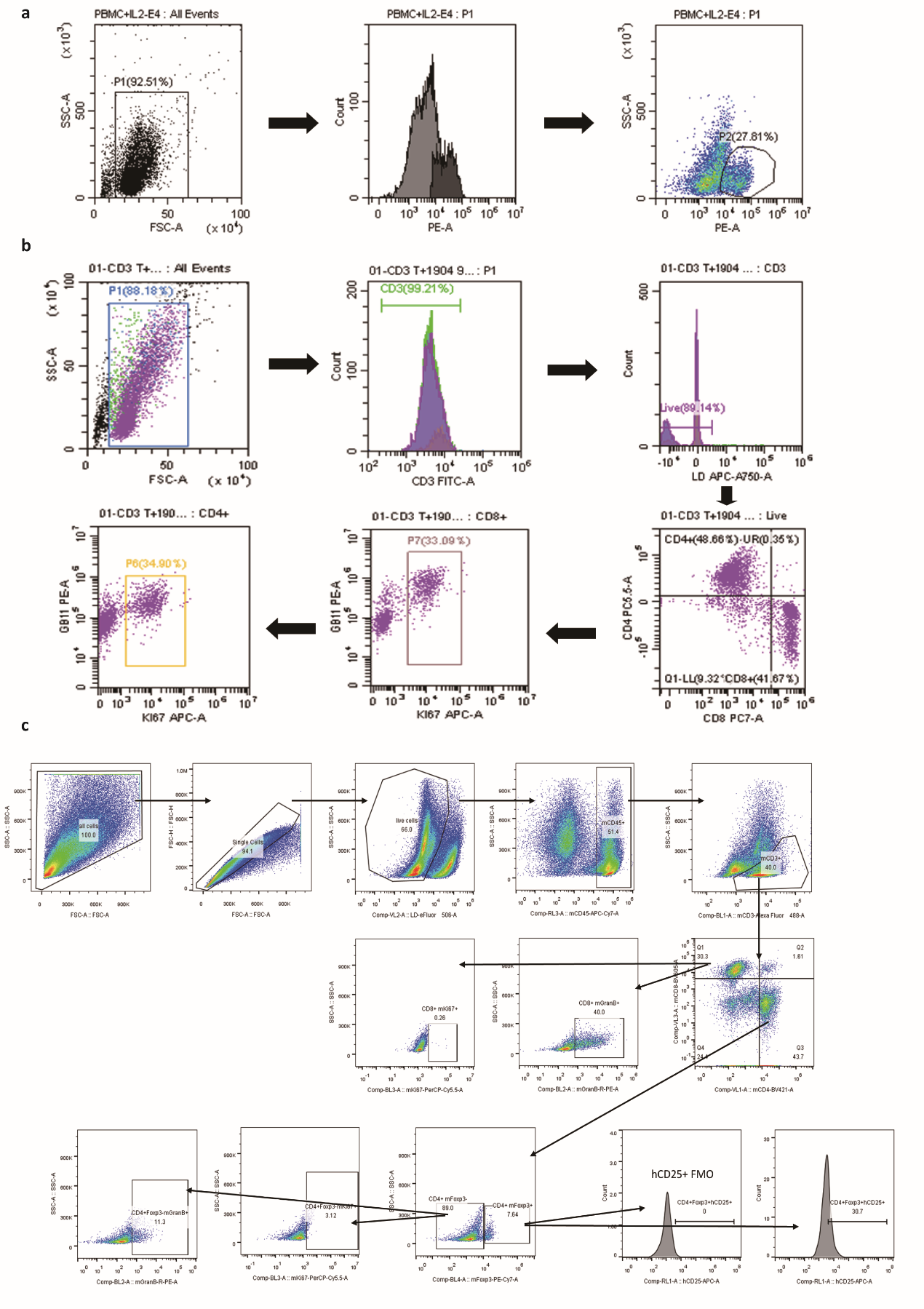
**

**Supplementary Figure S2.** The gating strategy for flow cytometry.

a, b, Gating strategy for IL-2/IL-2 receptor blocking activity in Figure 1 a, b.

c, Gating strategy for comparison of *in vivo* antitumor effects in Figure 7.


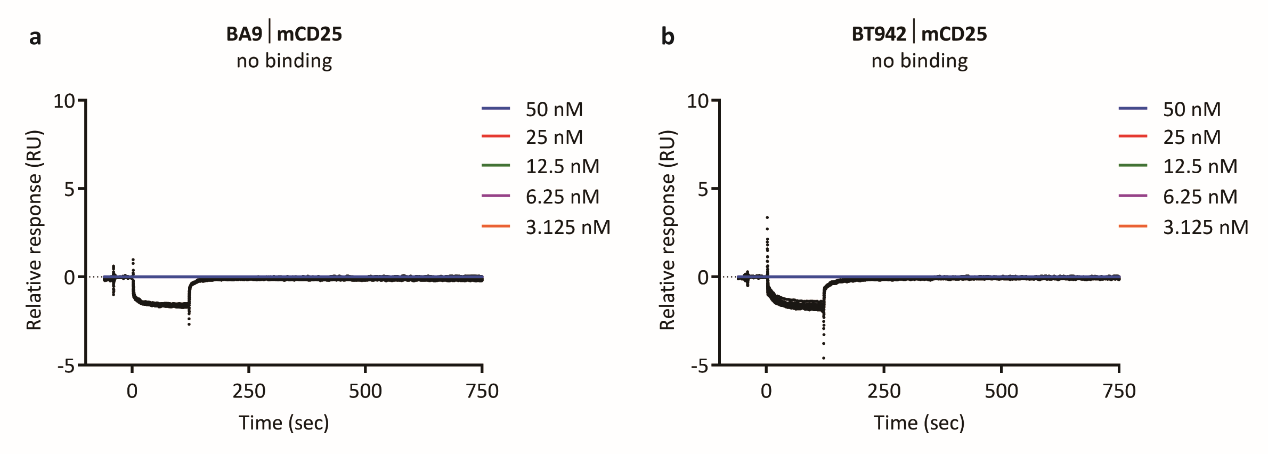


**Supplementary Figure S3.** Cross-reactivity with mouse CD25 by BA9 and BT942. Antibody was loaded onto the sensor, then serial dilutions of CD25 was injected. BA9 and BT942 cannot react with mouse CD25. Experiments were performed in duplicate.


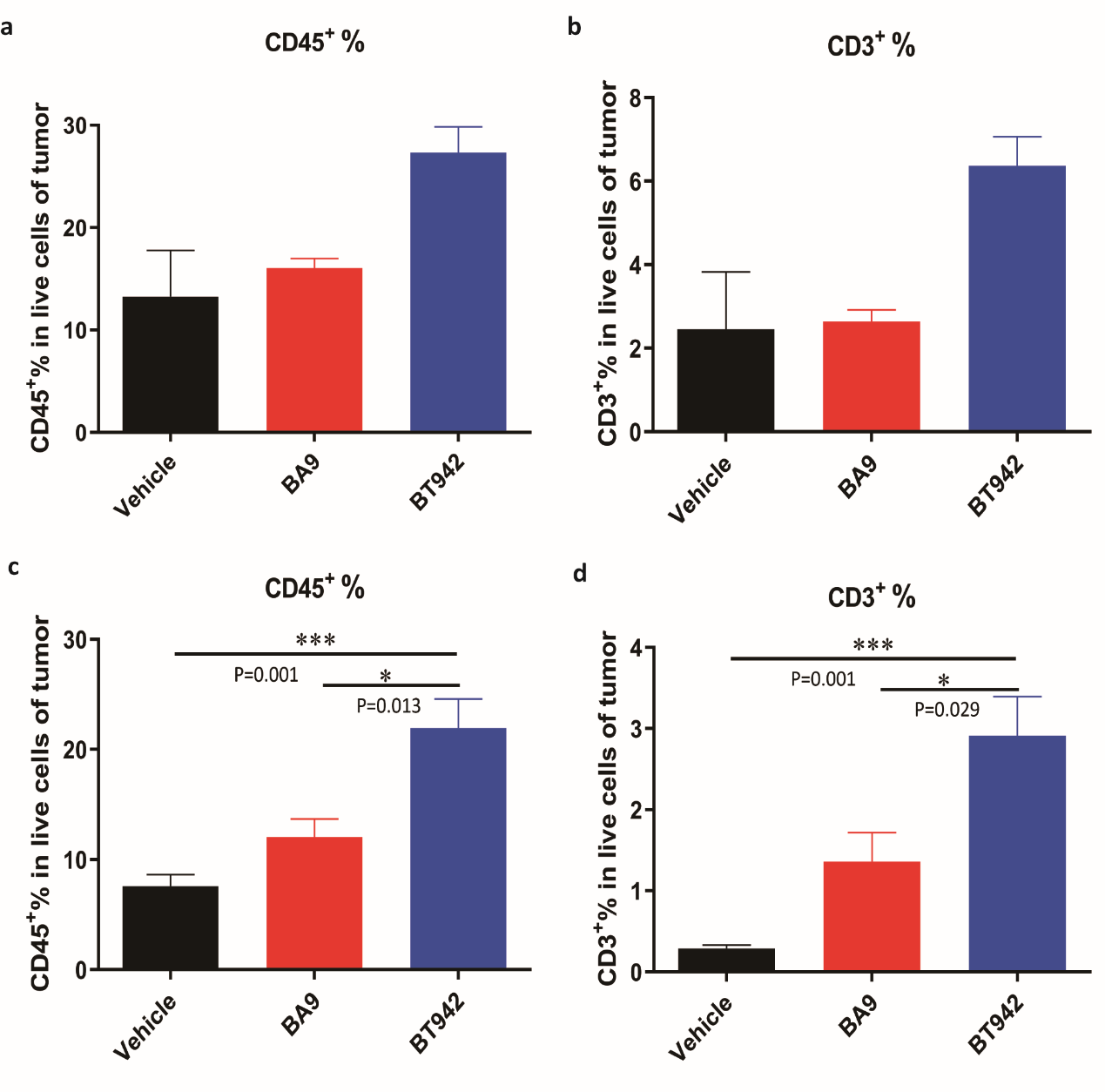


**Supplementary Figure S4.** Quantification of CD45^+^ and CD3^+^T population percentage in tumor cells from early (a, b) and late phase treatment (c, d). p values obtained by one-way ANOVA. Bars without labels indicate that no statistical significance was observed.


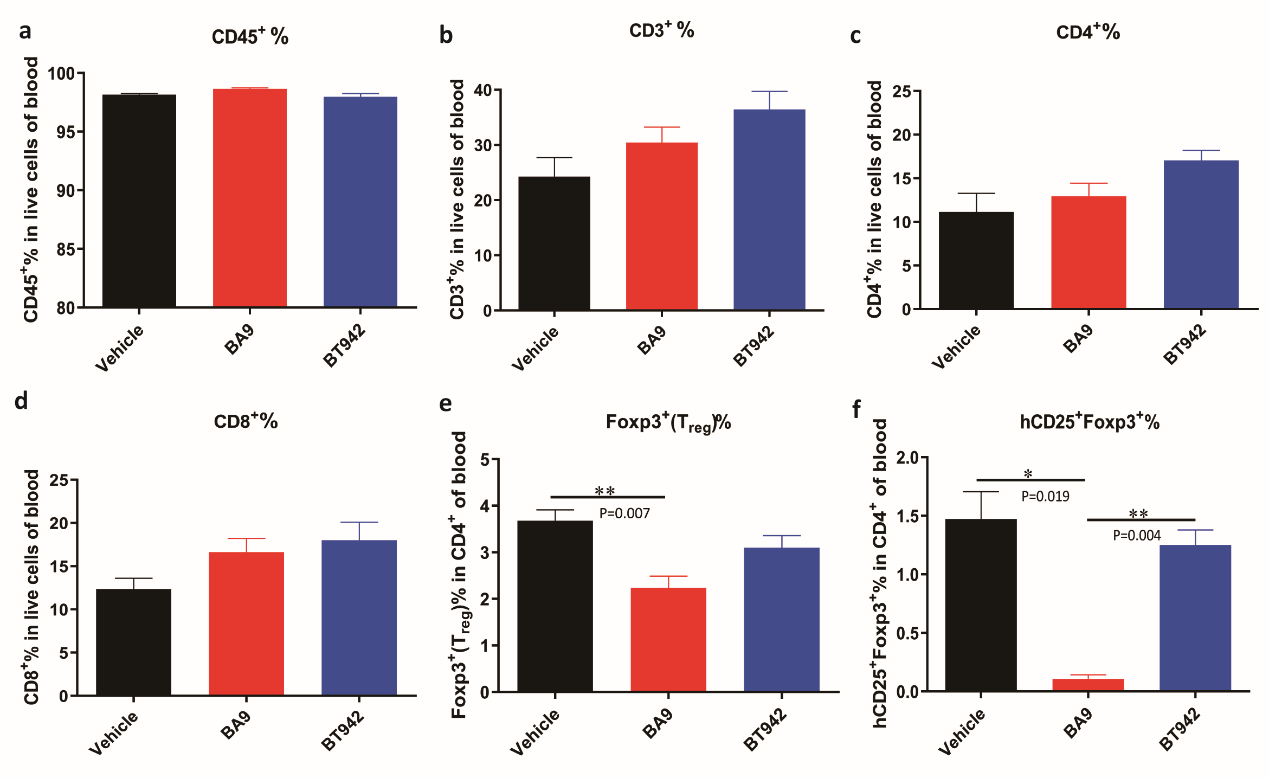


**Supplementary Figure S5.** Quantification of CD45^+^, CD3^+^, CD4^+^, CD8^+^, Foxp3^+^ (Treg) and hCD25^+^Foxp3^+^ population percentage in peripheral blood cells from late phase treatment. p values obtained by one-way ANOVA. Bars without labels indicate that no statistical significance was observed.

**
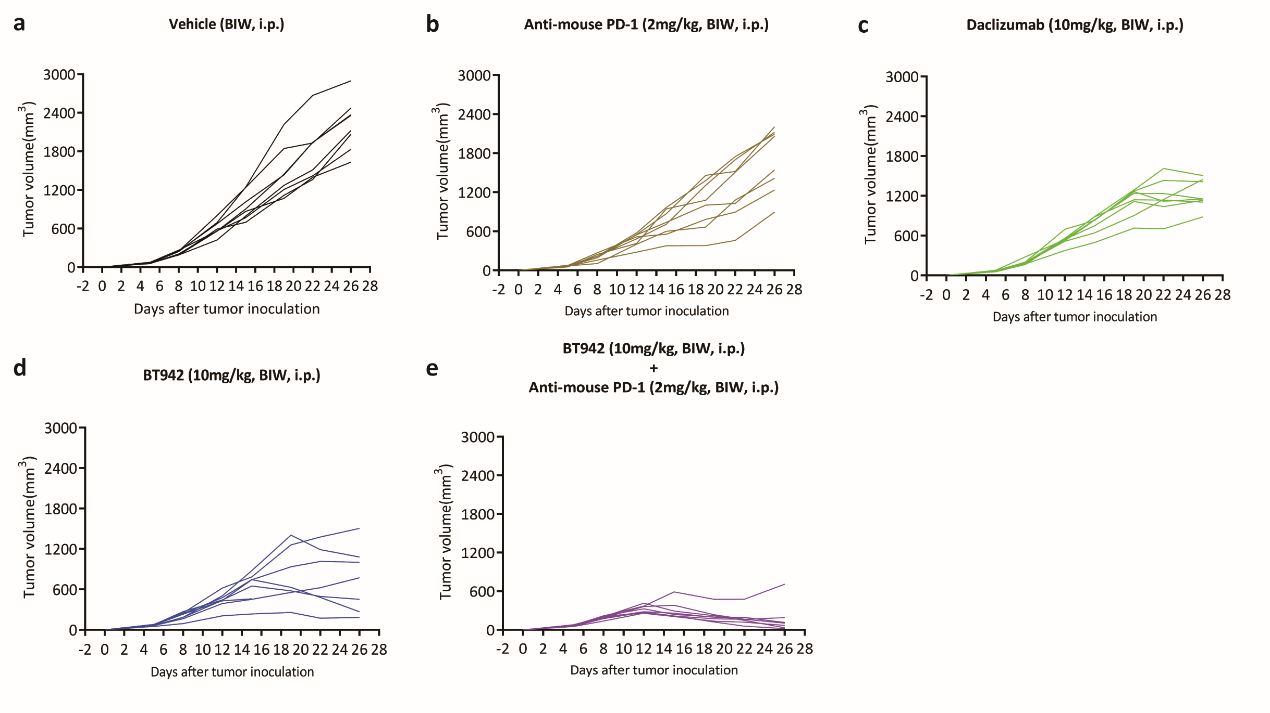
**

**Supplementary Figure S6.**  Individual data of tumor growth in mice treated with anti-mouse PD1 antibody, daclizumab, BT942 or combination of BT942 and anti-mouse PD1 (n=8).

**a.** Individual data of tumor growth in mice treated with vehicle control (black).

**b.** Individual data of tumor growth in mice treated with anti-mouse PD1 (brown).

**c.** Individual data of tumor growth in mice treated with Daclizumab (green).

**d.** Individual data of tumor growth in mice treated with BT942(blue).

**e.** Individual data of tumor growth in mice treated with BT942 and anti-mouse PD1 combination (purple).


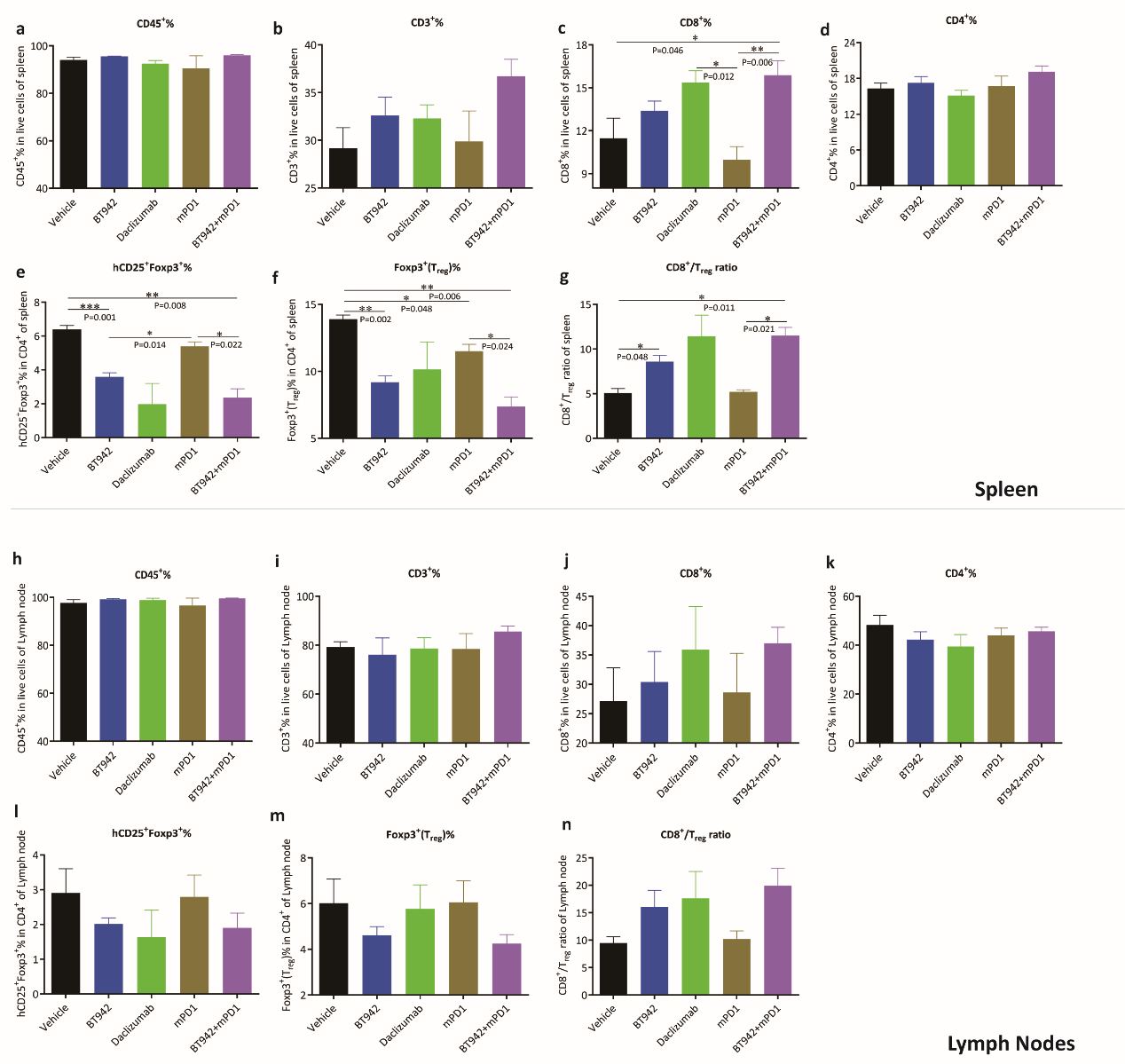


**Supplementary Figure S7.** Multiple immune cell populations in Spleen and Lymph Nodes were analyzed in flow cytometry after BT942, daclizumab and combination treatment in B-hIL2RA humanized mice. Cells in Spleen and Lymph Nodes (inguinal, axillary) were collected for flow cytometry analysis at day 26 after tumor inoculation.

**a, b, c, d, e, f.** Quantification of CD45^+^, CD3^+^, CD8^+^, CD4^+^T, hCD25^+^Foxp3^+^ and Foxp3^+^ (Treg) cell population percentage in live cells in Spleen. CD8^+^/Treg cell ratios were also analyzed.

**g, h, i, j, k.** Quantification of CD45^+^, CD3^+^, CD8^+^, CD4^+^T, hCD25^+^Foxp3^+^ and Foxp3^+^ (Treg) cell population percentage in live cells in Lymph Nodes (inguinal, axillary). CD8^+^/Treg cell ratios were also analyzed.

Data were presented as Mean ± SEM. p values were obtained by one-way ANOVA. Bars without labels indicate that no statistical significance was observed.


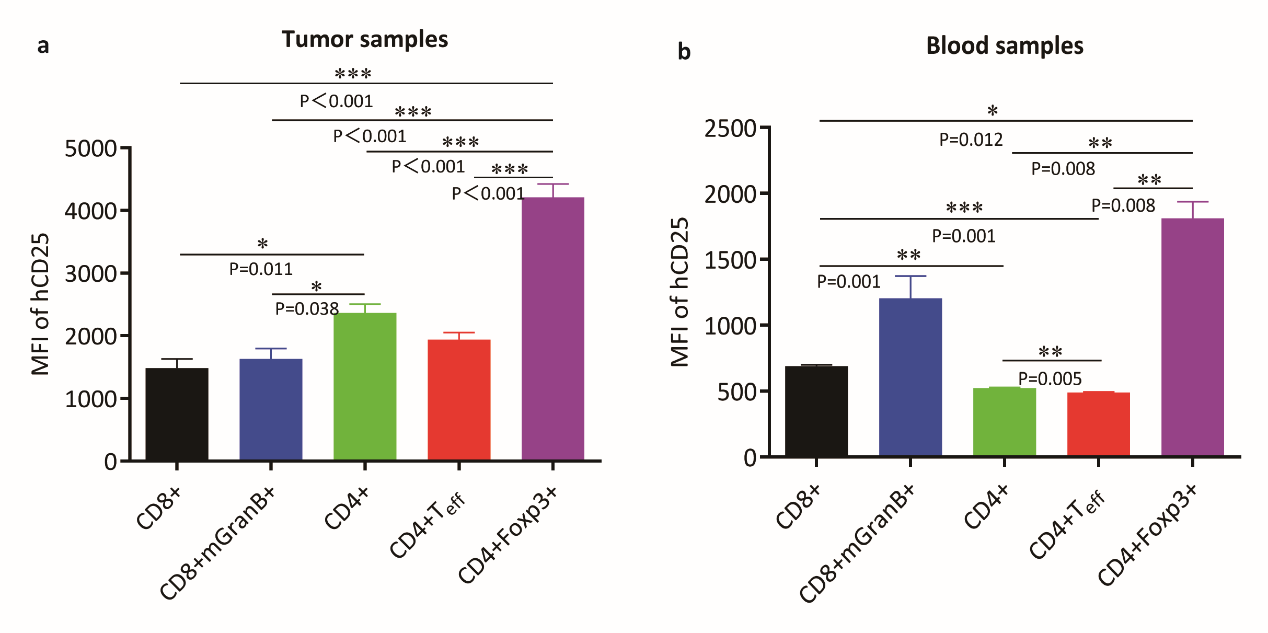


**Supplementary Figure S8.** Comparison of CD25 expression level in multiple immune cell populations from tumors and blood of mice in the vehicle group by flow cytometry.

1. CD25 expression levels were evaluated in mouse CD8^+^, CD8^+^ granzyme B^+^, CD4^+^, CD4^+^T_eff_ and T_reg_ (CD4^+^Foxp3^+^) cells from tumors of mice in the vehicle group at day 26 after tumor inoculation.
2. CD25 expression levels were evaluated in mouse CD8^+^, CD8^+^ granzyme B^+^, CD4^+^, CD4^+^T_eff_ and T_reg_ (CD4^+^Foxp3^+^) cells from blood of mice in the vehicle group at day 26 after tumor inoculation.

Cells were gated and analyzed by Attune NxT Flow Cytometer (Thermo Fisher). Data were presented as Mean ± SEM. p values were obtained by one-way ANOVA. Bars without labels indicate that no statistical significance was observed.

a b


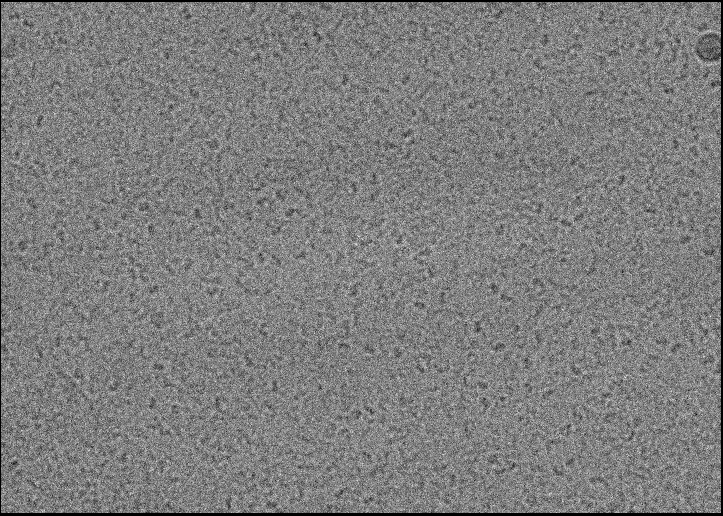

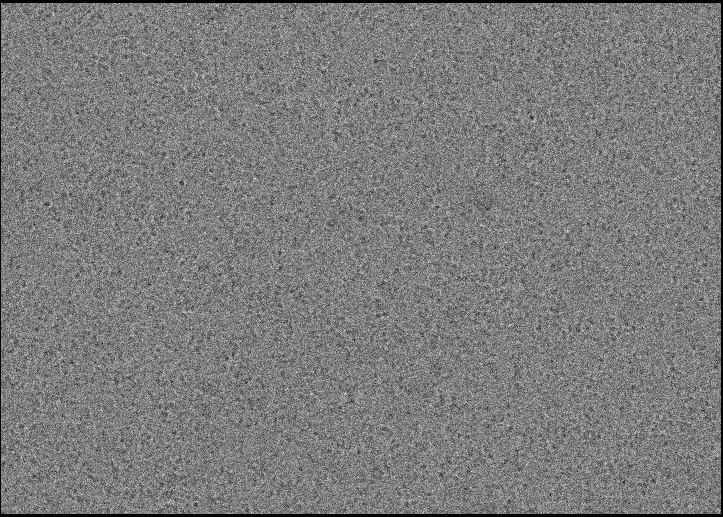


**c**

**
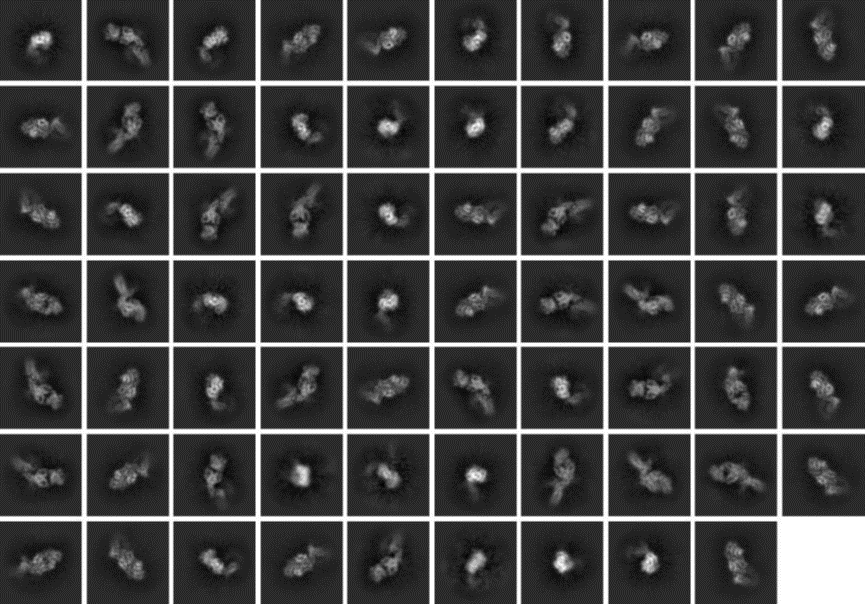
**

**Supplementary Figure S9.** Raw cryo-EM micrographs (a, b) and typical 2D class averages (c) of CD25-IL-2-BT942 Fab complex.

**Supplementary Table S1.** Mice, cell lines and reagents

| **REAGENT** | **IDENTIFIER** | **SOURCE** |
| --- | --- | --- |
| Human antibody transgenic mice | - | Shandong Boan Biotechnology |
| B-hIL2RA humanized mice | - | Biocytogen |
| Cynomolgus monkeys | - | Junke Zhengyuan (Beijing) Pharmaceutical Research |
| CD25/IL2RA Protein, Human, Recombinant (His Tag) | 10165-H08H | Sino Biological |
| Transcriptor First Strang cDNA Synthesis Kit | 4897030001 | Roche |
| SfiI | R0123L | New England Biolabs |
| TG1 competent cells | 60502-2 | Lucigen |
| PEG8000 | A600433-0500 | BBI Life Sciences |
| NaCl | 10019318 | Sinopharm |
| Dynabeads M-280 Streptavidin | 11206D | Invitrogen |
| Ez-link Sulfo-NHS-LC-Biotin | 21335 | Thermo Fisher Scientific |
| Stripwell Microplate | 42592 | Costar |
| High binding plates | 40301 | Beaver |
| Skim milk powder | 232100 | BD Biosciences |
| PBS | P1010 | Solarbio |
| Tween-20 | T8220 | Solarbio |
| Affinity purified antibody peroxidase Labeled Goat anti-human IgG(H+L) | 474-1006 | KPL |
| Streptavidin/HRP | 890803 | R&D Systems |
| FITC-anti human IgG Fc mAb | 409310 | Biolegend |
| CM5 chip | BR-1005-30 | GE healthcare |
| HBS-EP+ buffer | BR-1006-69 | GE healthcare |
| Human antibody capture kit | 29-2346-00 | GE healthcare |
| SA biosensors | 18-5019 | ForteBio |
| Expi-CHO Expression system | A29133 | Gibco |
| AT Protein A Diamond | AA0272 | Bestchrom |
| Jurkat | G7011 | Promega |
| SU-DHL-1 | CRL-2955 | ATCC |
| HEK293T-CD25 | - | KYinno |
| PBMC | PB003F-C | ALLCELLS |
| CD8+T cell | PB009-3-C | ALLCELLS |
| CD4+T cell | PB009-2F-C | ALLCELLS |
| Treg | PB009-4F-C | ALLCELLS |
| FITC anti-human CD3 | 317306 | Biolegend |
| PerCP/Cyanine5.5 anti-human CD4 | 357428 | Biolegend |
| PE/Cyanine7 anti-human CD8 | 344750 | Biolegend |
| PE anti-human CD25 | CD25-P-100 | BD |
| APC anti-human CD8Foxp3 | SPC-1323D-APC | Biolegend |
| CD3/CD28 active | 10971 | Stemcell |
| 2-mercaptoethanol | M6250 | Sigma |
| 1%Glutamax | 35050061 | Gibco |
| Sodium pyruvate | 2500-CIB | Gibco |
| X-VIVO | 01-418Q | LONZA |
| HEPES | 25-060-Cla | Gibco |
| Nonessential aminoacids | 11140050 | Thermo fisher |
| Rapamycin | V900930-1MG | Sigma |
| TGF-β | abs04204 | Absin |
| PE anti-STAT5 Phospho (Tyr694) | #936904 | Biolegend |
| APC anti-human Ki-67 | 350514 | Biolegend |
| PE anti-human/mouse Granzyme B Recombinant Antibody | 396406 | Biolegend |
| Foxp3 Fix/Perm buffer Set | 421403 | Biolegend |
| IL2 | abs00804 | Absin |
| Bio-Glo Luciferase Assay Buffer/Substrate | G7940 | Promega |
| Anti-mouse PD1 antibody | BE0146 | Bio X cell |
| Zombie NIR™ Fixable Viability Kit(DMSO) | 423106 | Biolegend |
| Anti-mouse CD16/32 | 101302 | Biolegend |
| Solarbio PBS buffer | P1020 | Solarbio |
| Tumor Dissociation Kit,mouse | 130-096-730 | Miltenyi Biotec |
| Brilliant Violet 510™ anti-mouse CD45 | 103138 | Biolegend |
| Alexa Flour® 488 anti-mouse CD3 | 100210 | Biolegend |
| Brilliant Violet 421™ anti-mouse CD4 | 100438 | Biolegend |
| Brilliant Violet 711™ anti-mouse CD8a | 100748 | Biolegend |
| APC anti-human CD25 | 302610 | Biolegend |
| PE/Cy™ 7 anti-mouse/rat Foxp3 | 25-5773-82 | eBioscience |
| Tumor Dissociation Kit,mouse | 130-096-730 | Miltenyi Biotec |
| RPMI Medium 1640（1X） | 11875-093 | gibco |
| Solarbio PBS Buffer | P1020 | Solarbio |
| Red Blood Cell Lysis Buffer | C3702 | Beyotime |
| Distilled water | 15230-162 | gibco |
| Purified anti-mouse CD16/32 | 101302 | Biolegend |
| Fixable Viability Dye eFluor ™506 | 65-0866-14 | eBioscience |
| APC/Cy7 anti-mouse CD45 | 103116 | Biolegend |
| Alexa Flour® 488 anti-mouse CD3 | 100210 | Biolegend |
| Brilliant Violet 421™ anti-mouse CD4 | 100438 | Biolegend |
| Brilliant Violet 605™ anti-mouse CD8a | 100744 | Biolegend |
| APC anti-human CD25 Antibody | 302610 | Biolegend |
| Anti-Mo/Rt Foxp3PE/Cy™ 7 | 25-5773-82 | eBioscience |
| PerCP/Cy5.5 anti-mouse Ki-67 | 652424 | Biolegend |
| anti-h/m Granzyme B-PE | 372208 | Biolegend |
| APC Mouse IgG1,K Isotype Ctrl | 400120 | Biolegend |
| PE/Cy™ 7 Rat IgG2a K Isotype Ctrl | 25-4321-82 | eBioscience |
| PerCP/Cy5.5 Rat IgG2a,K Isotype Ctrl | 400532 | Biolegend |
| PE Mouse IgG1, k isotype Ctrl | 400112 | Biolegend |
| Foxp3/Transcription Factor Staining Buffer Set | 00-5523-00 | eBioscience |

**Supplementary Table S2.** The average tumor volume ± SEM and the Tumor Growth Inhibition value per group.

| **Sample** | **Tumor volume(mm^3^) AVG±SEM** | **TGI(%)** |
| --- | --- | --- |
| Vehicle | 2221±140 | - |
| Anti-mouse PD-1 | 1698±174 | 24.3 |
| BT942 | 753±181 | 68.2 |
| BT942+ Anti-mouse PD-1 | 171±80 | 95.2 |

**Supplementary Table S3.**  PK parameters of single intravenous administration of BT942 in cynomolgus monkeys (n=2).

| **Parameter** | **Unit** | **BT942** |
| --- | --- | --- |
| T_1/2_ | hours | 206.97±19.03 |
| T_max_ | hours | 0.13±0.16 |
| C_max_ | μg/mL | 316.51±17.48 |
| C_0_ | μg/mL | 316.23±17.06 |
| AUC_(0-t)_ | hours*μg/mL | 32036.89±1234.97 |
| AUC_(0-∞)_ | hours*μg/mL | 38548.79±1874.86 |
| Vz/F | mL/kg | 77.38±3.36 |
| CLz/F | mL/hour/kg | 0.26±0.01 |
| MRT_(0-t)_ | hours | 273.18±6.83 |

Footnote:

t_1/2_ Elimination half-life

T_max_ Peak Time

C_max_ Peak Concentration

C0 Initial Concentration

AUC_(0-t)_ Area under the curve from the time of dosing to the last measurable concentration

AUC_(0-∞)_ Area under concentration-time curve 0-∞

Vz/F The volume of distribution

CLz/F Clearance

MRT_(0-t)_ Mean residence time

**Supplementary Table S4. Cryo-EM data collection, refinement, and validation statistics**

|  | CD25-Fab complex map  (EMD-31499) |
| --- | --- |
| **Data collection and processing** |  |
| Magnification | 130k |
| Voltage (kV) | 300 |
| Electron exposure (e^–^/Å^2^) | 50 |
| Defocus range (μm) | -1.4~-2.4 |
| Pixel size (Å) | 0.54 |
| Symmetry imposed | C1 |
| Initial particle images (no.) | 1,864,500 |
| Final particle images (no.) | 149,373 |
| Map resolution (Å)  FSC threshold | 3.2  0.143 |
| Map resolution range (Å) | 3.0-10.0 |
|  |  |
| **Refinement** |  |
| Initial model used (PDB code) | 6YIO |
| Map sharpening *B* factor (Å^2^) | -90.0 |
| Model composition  Non-hydrogen atoms  Protein residues  Ligands | 4217  547  0 |
| *B* factors (Å^2^)  Protein  Ligand |  |
| R.m.s. deviations  Bond lengths (Å)  Bond angles (°) | 0.005  0.700 |
| Validation  MolProbity score  Clashscore  Poor rotamers (%) | 1.80  9.16  0.63 |
| Ramachandran plot  Favored (%)  Allowed (%)  Disallowed (%) | 95.53  4.47  0.00 |
